# Somatic influences on subjective well-being and affective disorders: the convergence of thermosensory and central serotonergic systems

**DOI:** 10.3389/fpsyg.2014.01580

**Published:** 2015-01-13

**Authors:** Charles L. Raison, Matthew W. Hale, Lawrence E. Williams, Tor D. Wager, Christopher A. Lowry

**Affiliations:** ^1^Department of Psychiatry, Norton School of Family and Consumer Sciences, College of Medicine, College of Agriculture and Life Sciences, University of ArizonaTucson, AZ, USA; ^2^Department of Psychology, School of Psychological Science, La Trobe UniversityBundoora, Australia; ^3^Marketing Division, Leeds School of Business, University of Colorado BoulderBoulder, CO, USA; ^4^Department of Psychology and Neuroscience, University of Colorado BoulderBoulder, CO, USA; ^5^Department of Integrative Physiology, University of Colorado BoulderBoulder, CO, USA

**Keywords:** embodied cognition, interpersonal warmth, lateral parabrachial nucleus, raphe, serotonin, spinoparabrachial, spinothalamic, warm temperature

## Abstract

Current theories suggest that the brain is the sole source of mental illness. However, affective disorders, and major depressive disorder (MDD) in particular, may be better conceptualized as brain-body disorders that involve peripheral systems as well. This perspective emphasizes the embodied, multifaceted physiology of well-being, and suggests that afferent signals from the body may contribute to cognitive and emotional states. In this review, we focus on evidence from preclinical and clinical studies suggesting that afferent thermosensory signals contribute to well-being and depression. Although thermoregulatory systems have traditionally been conceptualized as serving primarily homeostatic functions, increasing evidence suggests neural pathways responsible for regulating body temperature may be linked more closely with emotional states than previously recognized, an affective warmth hypothesis. Human studies indicate that increasing physical warmth activates brain circuits associated with cognitive and affective functions, promotes interpersonal warmth and prosocial behavior, and has antidepressant effects. Consistent with these effects, preclinical studies in rodents demonstrate that physical warmth activates brain serotonergic neurons implicated in antidepressant-like effects. Together, these studies suggest that (1) thermosensory pathways interact with brain systems that control affective function, (2) these pathways are dysregulated in affective disorders, and (3) activating warm thermosensory pathways promotes a sense of well-being and has therapeutic potential in the treatment of affective disorders.

## Thermosensory signaling in the context of embodied cognition

The proposition that mental illnesses are brain disorders has become a foundational principle guiding funding priorities at the National Institutes of Health. On the face of it, this seems an altogether reasonable course of action. After all, given the high likelihood that mental states arise from functional states of the brain, what other kind of disorders could mental illnesses be?

The answer to this apparently rhetorical question is that mental disorders in general—and major depressive disorder (MDD) in particular—may be embodied conditions that derive causality in non-trivial ways not just from the brain, but also from domains that lie outside the central nervous system (CNS). Indeed, significant research now suggests that human emotions, cognition and behavior arise not just from the activity of brain circuitry, but also from our sensory-motor experience in relation to multiple aspects of the environments in which we find ourselves. At its most radical, the concept of embodiment suggests—to quote a recent review—that “the brain is not the sole cognitive resource we have available to us to solve problems. Our bodies and their perceptually guided motions through the world do much of the work required to achieve our goals, replacing the need for complex internal mental representations” (Wilson and Golonka, 2013). A more modest claim, and the only one required to substantiate the argument of the current paper, is that emotional/cognitive states can be powerfully shaped by signals coming to the brain from the body, the larger environment, or both. In light of recent findings demonstrating antidepressant effects of peripheral-acting therapies (Raison et al., 2013), and the importance of the microbiota in cognitive function (Cryan and Dinan, 2012; Bested et al., 2013), disorders like depression are increasingly thought of in terms of interactions between peripheral systems and brain.

Although more modest versions of embodied cognition seem non-controversial enough, they are in fact potentially quite subversive, because they suggest that although the brain may always be the proximal cause of a mental state such as depression, it may often not be the initiating cause, or even the most important cause. So, for example, it may be that some patients have abnormalities in the structure of their brains that generate patterns of CNS activity that produce depressive cognitions and emotions irrespective of any positive or negative cues from either the body or the environment. This is likely to be true in many cases of post-stroke depression, for example. It might be truly said that in such patients MDD is a brain disorder. But consider other patients who receive depressogenic signals from the environment that drive CNS activity into patterns that promote depression without damaging the underlying structure or functional capacity of their brains. Showing that the termination of the depressogenic signal from the external environment ended the depression would make a strong argument that the brains of these people were responding normally to external stimuli, even though this response provoked depression. We say a “normal response” because if the depression was being caused by some damage intrinsic to the brain how could it be that removing an external cause would end the depression? Therefore, in these cases, it would seem that the most important cause of the condition is not the normal evolved brain response to the environmental stimulus, but the stimulus itself.

We begin our exploration of the role of thermosensory pathways in the pathogenesis and treatment of affective disorders with a simple question. If stimuli outside the brain can cause these conditions, might they also be harnessed to treat them? And if such factors exist, are some better targets than others? We think the answer to both questions is yes. And, consistent with the embodied perspective that cognitive processes can arise from an organisms' sensorimotor experience, we suggest that sensory pathways are especially promising candidates as antidepressant pathways. We have several reasons for this conviction. First, sensory pathways impact the brain powerfully because they evolved to be truth-tellers and thereby to accurately alert the organism to environmental factors of greatest significance to its current overall reproductive fitness. Second, sensory pathways, through multisynaptic connections, have specific CNS targets and tend to produce specific effects. Third, preliminary evidence suggests that activity in these pathways can profoundly impact the emotions, cognitions and behaviors that comprise depression.

Increasing evidence suggests that all sensory pathways are likely relevant to affective disorders (and especially depression) and their treatment, both the classic five senses (taste, touch, sight, sound, smell) and senses such as the sense of ambient light by intrinsically photosensitive retinal ganglion cells (ipRGCs), kinesthetic sense, pain, temperature, and immune signaling that function to report on the internal state of the body. However, in this paper we focus specifically on thermosensory pathways and their CNS targets, based on increasing evidence that they hold significant promise, both in deepening our understanding of the biology of MDD and in enhancing our capacity to treat it. We commence by describing the integrated circuitry by which peripheral thermal information is conveyed to, and processed by, the CNS. We then review evidence linking thermosensory phenomena and biology (both peripheral and central) with affective states in general and with depression in particular. Following this we provide evidence that thermosensory pathways function abnormally in MDD. We conclude with recent evidence—based on studies using whole-body hyperthermia (WBH)—that peripheral thermosensory pathways may hold promise as novel treatment modalities for MDD.

## Brain-body circuitry linking non-noxious thermosensation to affect

### Overview

This section focuses on pathways by which thermal information is transferred from the periphery to the CNS, and how—within the CNS—different aspects of thermosensory information are processed. We focus on non-noxious thermal stimuli because, as we discuss at several junctures, these appear to be most relevant to both the pathophysiology and treatment of mood, and perhaps anxiety, disorders. In this section we first trace afferent thermosensory pathways to the CNS, then discuss brain regions involved in the various aspects of thermal perception and conclude by reviewing evidence that these brain regions function abnormally in individuals with mood disorders and may be restored to more appropriate activity levels following antidepressant treatment.

### Mechanisms underlying non-noxious thermal signaling to the CNS

In this section we explore mechanisms through which cutaneous warmth signals to the CNS. It is important to recognize the difference and often complete dissociation between skin temperature and core body temperature and their functional relation. Skin temperature and core body temperature are functionally interrelated and vary in a diurnal pattern. Sleep typically occurs during a circadian phase of increased heat loss, which is due to both a decrease in heat production as well as an increase in skin blood flow and the associated skin warming and transfer of heat to the environment (Van Marken Lichtenbelt et al., 2006; Van Someren, 2006). Under natural living conditions, both proximal and distal skin temperature increase substantially at the onset of the inactive period and remain elevated until early morning, when they fall precipitously. The increases in proximal and distal skin temperatures at the onset of the inactive period result in heat loss and coincide with a decline in core body temperature. These increases in skin temperature causally increase sleep propensity (Van Marken Lichtenbelt et al., 2006; Van Someren, 2006), presumably through activation of the afferent neural pathways outlined in this paper projecting to neural systems controlling sleep. As it is cutaneous warmth that drives thermoregulatory responses (Nakamura and Morrison, 2010) and sleep physiology, here we focus on mechanisms through which signals of cutaneous warmth are relayed to the CNS.

Thermal signals are relayed to the CNS via primary thermosensors in the cutaneous membrane, to secondary thermosensory neurons in lamina I of the spinal (and trigeminal) dorsal horn (Craig, 2003). Somatosensory neurons located in lamina I of the spinal dorsal horn are thought to fall into three different categories: (1) nociception-specific neurons that respond to noxious mechanical and heat stimuli, (2) polymodal nociceptive neurons that respond to noxious mechanical, heat, and cold stimuli, and (3) thermoreception-specific neurons that respond linearly to graded, innocuous warming or cooling stimuli and are not further activated by temperatures in the noxious range (Andrew and Craig, 2001; Craig et al., 2001; Craig, 2003). A fourth type of somatosensory neuron in lamina I of the spinal dorsal horn, responsive to affective touch, will be discussed in detail later.

Given the distinction between nociceptive and non-nociceptive thermal sensory signaling, even at the level of lamina I of the spinal dorsal horn, we focus in this review on thermal signals within the non-noxious range. There are many reasons to restrict the focus. For example, the molecular mechanisms of non-noxious warm signaling (e.g., TRPv4) are different from noxious heat (e.g., TRPv1) (Patapoutian et al., 2003) and the primary sensory neurons are different (McCleskey, 1997). Consequently, nociceptive heat is not simply an extension of non-nociceptive warmth. This is clear in that warmer temperatures are associated with increasing positive affect, but this is clearly not the case when pain thresholds are reached. Painful heat is not “pleasant” in the way that warm temperature is. Because we believe that there is an intrinsic relationship between non-noxious “warmth,” thermoregulatory function, serotonergic systems, and affect/well-being, it makes sense to restrict our focus to the non-nociceptive range of temperatures. From an evolutionary perspective it also makes sense to focus on “non-noxious” warmth, given that warm temperature is intrinsically rewarding because it reduces the metabolic cost of maintaining core body temperature. Thus, vertebrates demonstrate motivational behavior in pursuit of environmental warmth (Terrien et al., 2011), including the pursuit of physical contact with conspecifics (a behavior known as “kleptothermy”) (Brischoux et al., 2009). On the other hand, noxious heat, like other danger signals, prompts behavioral withdrawal and activates other brain-body pathways associated with the stress response and negative emotionality. Finally, as we discuss later in this paper, the administration of non-noxious warmth via WBH shows promise as an antidepressant strategy, whereas nociceptive stimuli coming from the periphery—especially when chronic—are profoundly depressogenic (Hilderink et al., 2012).

**Figure 1:**
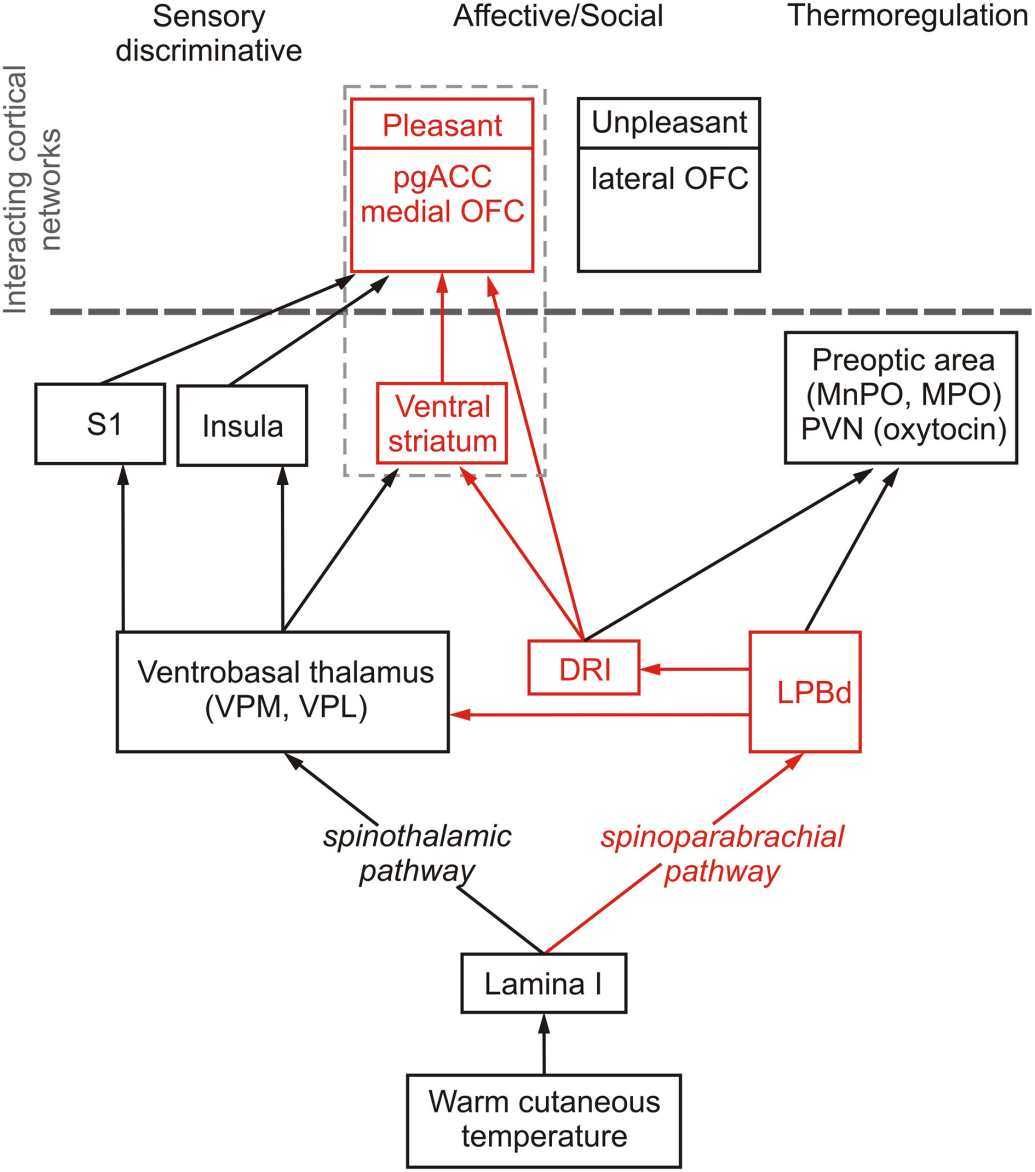
**Schematic diagram illustrating sensory discriminative, thermoregulatory and affective/social (shown in red) pathways mediating responses to warm cutaneous temperature**. Temperature-sensitive proteins expressed in the skin respond to warm temperature and, via spinal projection neurons in lamina I of the spinal dorsal horn, signal to the brain through spinothalamic and spinoparabrachial pathways that control sensory discriminative, affective/social and thermoregulatory responses to warm cutaneous temperature. The spinothalamic pathway consists of projections from lamina I spinal neurons to thermosensitive neurons in the ventrobasal thalamus, including the ventral posteromedial nucleus (VPM) and ventral posterolateral nucleus (VPL) which project to the insula (Augustine, 1996), the thermosensory cortex mediating sensory discriminative functions (Craig et al., 1994, 1999), and somatosensory cortex where thermal stimuli are represented (Rolls et al., 2008), which project to the orbitofrontal cortex and anterior cingulate cortex (Carmichael and Price, 1995; Augustine, 1996). The spinoparabrachial pathway transmits warm cutaneous stimuli via the dorsal part of the lateral parabrachial nucleus (LPBd) to hypothalamic regions associated with thermoregulation, including the medial preoptic area (MPO) and median preoptic nucleus (MnPO), which control physiological and behavioral thermoregulatory responses to cutaneous warmth (Nakamura and Morrison, 2010), and the paraventricular nucleus of the hypothalamus (PVN) (Ziegler et al., 2012), which may account for the effect of warm temperature to increase release of oxytocin (Uvnas-Moberg et al., 1993), a neuropeptide with well-documented anxiolytic and prosocial/affiliative effects (Ross and Young, 2009; Yoshida et al., 2009; Rilling and Young, 2014), that can, by itself, induce thermoregulatory cooling and hypothermia (Hicks et al., 2014). Alongside the thermoregulatory pathway, the spinoparabrachial pathway also signals via the LPBd and multisynaptic pathways to brainstem and forebrain regions associated with affective and social aspects of warm cutaneous temperature. The affective/social thermosensory systems consist of an interconnected network arising from the LPB, which projects to the dorsal raphe nucleus, interfascicular part (DRI; Saper and Loewy, 1980; Lee et al., 2003), including DRI serotonergic neurons. Warm afferent thermal signals are subsequently relayed to forebrain structures implicated in the affective component of thermal responses, e.g., pregenual cingulate cortex (pgACC) (Porrino and Goldman-Rakic, 1982), medial orbitofrontal cortex (medial OFC) (Porrino and Goldman-Rakic, 1982), and ventral striatum (Van Bockstaele et al., 1993).

The classic non-noxious thermosensory pathway consists of a crossed lateral spinothalamic tract from lamina I of the dorsal horn to the ventral thalamus (Figure 1), specifically the ventral posteromedial nucleus (VPM) (Craig et al., 1994, 1999; Dostrovsky and Craig, 1996; Blomqvist et al., 2000; Gauriau and Bernard, 2004; Zhang et al., 2006).

Functional anatomical studies in primates suggest that the thalamus relays, in a topographically organized manner, discriminative non-noxious thermosensory-specific lamina I spino- and trigeminothalamic projections to the dorsal region of the contralateral middle/posterior insular cortex (Craig et al., 1994, 1999). The role of the contralateral middle/posterior insular cortex in discrimination of the intensity of thermal stimuli within non-nociceptive and nociceptive ranges has been supported by other studies (Casey et al., 1996; Craig et al., 2000; Maihofner et al., 2002; Hua et al., 2005; Stancak et al., 2006; Rolls et al., 2008; Wager et al., 2013; Atlas et al., 2014). Patients with stroke damage in this region have post-stroke central pain and selective thermosensory dysfunction, without other somatosensory abnormalities (Cattaneo et al., 2007; Garcia-Larrea et al., 2010). Thus, the contralateral middle/posterior insular cortex represents the primary discriminative thermosensory representation of innocuous temperature, i.e., discriminating differences in stimulus intensity and location of innocuous temperature sensation in the human brain. In other words, the human thermosensory cortex is located in the insular cortex.

In addition to discriminative properties, non-noxious thermosensory signals also have thermoregulatory functions. Recent studies in rats have localized thermosensory pathways involved in homeostatic thermoregulatory control. These thermoregulatory pathways involve projections from lamina I of the dorsal horn to the lateral parabrachial nucleus (LPB) in the brainstem (Nakamura and Morrison, 2010), which seem to parallel pathways previously implicated in nociceptive signaling (Bushnell et al., 2013) (Figure 1). Interestingly, the thermoregulatory function of afferent thermal signals (e.g., as defined by projections to the preoptic area, the thermoregulatory command center of the brain) are independent of the spinothalamic pathway. Innocuous *cold* signals arising from lamina I of the dorsal horn excite glutamatergic projection neurons in the external lateral part of the lateral parabrachial nucleus (LPBel) that project to the preoptic area to mediate sympathetic and shivering thermogenic responses and also metabolic and cardiac responses (Nakamura and Morrison, 2008). In contrast, innocuous *warm* signals arising from lamina I of the dorsal horn excite glutamatergic projection neurons in the dorsal part of the lateral parabrachial nucleus (LPBd) that also project to the preoptic area and play a role in heat defense (thermoregulatory cooling) (Nakamura and Morrison, 2010). Thus, even at the level of the brainstem cold-sensitive and warm-sensitive pathways implicated in thermoregulatory homeostasis are discrete and divergent. These findings are consistent with evidence for both cold sensitive and warm sensitive lamina I neurons (Christensen and Perl, 1970; Dostrovsky and Hellon, 1978; Dostrovsky and Craig, 1996; Han et al., 1998). Furthermore, these findings are consistent with activation of a brainstem region in human volunteers, presumed to be either the periaqueductal gray or the parabrachial nucleus, that is correlated with both stimulus temperature and volunteers' subjective ratings of thermal stimulus intensity in the non-nociceptive (Craig et al., 2000) and nociceptive ranges (Tracey and Iannetti, 2006; Buhle et al., 2013). Future studies utilizing methods for identification of discrete functional subregions of the human periaqueductal gray (Satpute et al., 2013) should improve our understanding of the role of this region in thermosensory signaling and homeostatic thermoregulatory control. Cortical structures implicated in thermoregulatory responses, based on connectivity with the preoptic area during cutaneous warming, include the anterior midcingulate (dorsal) cortex and the right anterior insula (Figure 1) (Farrell et al., 2014).

A third property of non-noxious thermosensory signaling is its affective component. Craig et al. (2000) hypothesized that the affective component of an innocuous thermal stimulus, which is dependent on thermoregulatory integration (in contrast to the discriminative component, which is not) could arise from contextual evaluation in the right anterior insula or orbitofrontal cortex. In contrast, pain affect has been more closely associated with the dorsal anterior cingulate cortex (Rainville et al., 1997). A study designed to dissociate the discriminative aspect of non-noxious thermosensation from the affective aspect was conducted by Rolls et al. (2008). Consistent with Craig and colleagues' predictions, activation of the mid-orbitofrontal cortex, as well as the pregenual anterior cingulate cortex and the ventral striatum, was correlated with subjective pleasantness ratings made to warm (41°C) and cold (12°C) stimuli, and combinations of warm and cold stimuli, applied to the hand (Rolls et al., 2008) (Figure 1). The same structures have been associated with pleasant oral temperature and pleasant touch (Rolls et al., 2003b; Guest et al., 2007). Consistent with the discrete and divergent nature of warm and cold sensory signals apparent even at the level of the brainstem LPBd/LPBel, unpleasantness of cold stimuli was correlated with activation in different cortical structures, specifically the lateral and more anterior parts of the orbitofrontal cortex (Rolls et al., 2008). Connectivity of the preoptic area mediating thermoregulatory responses to cutaneous warmth has been associated with activity in the anterior midcingulate (dorsal) cortex, a region implicated in autonomic control, including control of sweating (Critchley et al., 2011), and the right anterior insula (Farrell et al., 2014). The dorsal anterior cingulate cortex is also associated with anhedonia (Wacker et al., 2009) and the anti-anhedonic effects of ketamine, a novel, rapid-acting antidepressant (Lally et al., 2014), reinforcing potential links between warm-sensitive neural systems and affective disorder.

### Brain regions relevant to non-noxious warmth, cold and the larger domain of pleasant and unpleasant sensations

#### The medial-orbitofrontal and pregenual cingulate cortical regions respond to diverse modalities of pleasant sensations

In addition to sensation of pleasant warm cutaneous stimuli, representations of a number of other pleasant stimuli are described in the medial-orbitofrontal cortex and pregenual cingulate cortex. These include oral warmth (Guest et al., 2007), the attractiveness of a face (O'Doherty et al., 2003; Kranz and Ishai, 2006) (see also Kringelbach and Rolls, 2004; Rolls, 2005, 2009), pleasant flavor (Small and Prescott, 2005; McCabe and Rolls, 2007; Rolls and McCabe, 2007; Small, 2012), pleasant odor (Gottfried et al., 2002; Rolls et al., 2003a, 2008; Grabenhorst et al., 2007), pleasant taste (Grabenhorst and Rolls, 2008), pleasant touch (Rolls et al., 2003b; McCabe et al., 2008; Rolls, 2010), visual images of chocolate (McCabe et al., 2012), sexual arousal (Redoute et al., 2000; Karama et al., 2002; Ponseti et al., 2006), successfully completing a biofeedback task (Nagai et al., 2004), winning money (Breiter et al., 2001; O'Doherty et al., 2001), and willingness to pay in economic transactions (Plassmann et al., 2007). Single unit recordings in primates demonstrate both orbitofrontal cortex neurons that respond to temperature only, or to temperature and/or taste and/or viscosity and/or gritty texture and/or fat texture (Kadohisa et al., 2004). Thus, there is specificity to the processing of different stimuli, including temperature, at the level of the medial orbitofrontal cortex, but as a whole this region generalizes to diverse modalities of pleasant stimuli.

#### The lateral orbitofrontal cortex responds to diverse modalities of unpleasant sensations

Meta-analytic connectivity modeling reveals distinct functional connectivity of the medial and lateral orbitofrontal cortices (Zald et al., 2014). Whereas, as discussed above, the medial orbitofrontal cortex has been shown to correlate with the pleasantness of a non-noxious thermal stimulus, activation of the lateral orbitofrontal cortex has been shown to correlate with the unpleasantness of a non-noxious thermal stimulus (Rolls et al., 2008). In the study by Rolls and colleagues, the coldest stimulus, which was rated the most unpleasant, resulted in the greatest activation of lateral orbitofrontal cortex, while the warmest stimulus, which was rated the most pleasant, resulted in the greatest deactivation of this area. In addition to unpleasantness related to cold temperature, unpleasantness related to other stimuli has also been associated with activation of the lateral orbitofrontal cortex. These stimuli include unpleasant odor (O'Doherty et al., 2001; Gottfried et al., 2002; Rolls et al., 2003a), unpleasant images (McCabe et al., 2012), unpleasant touch (Rolls et al., 2003b), cognitive modulation of affective touch (McCabe et al., 2008), and losing money (Kringelbach and Rolls, 2004; Rolls, 2009). In contrast, affect associated with noxious thermal stimuli has been more clearly associated with the anterior cingulate cortex (Rainville et al., 1997), suggesting a possible dissociation between systems encoding affective responses to non-noxious but unpleasant (cold) and noxious thermal (heat) stimulation.

#### How does information about skin temperature reach brain regions involved in positive affective responses to warm temperature?

It has been proposed that information about skin temperature may reach the medial orbitofrontal cortex through the somatosensory cortex and insular somatosensory areas (Rolls et al., 2008), both of which are known to project to the medial orbitofrontal cortex (Carmichael and Price, 1995). Nevertheless, the specific pathways through which information about skin temperature reaches the medial orbitofrontal cortex, including the relative contributions of the spinothalamic and spinoparabrachial pathways, is not clear and identification of these pathways will require further study. An alternative hypothesis is that warm signals are relayed to the medial orbitofrontal cortex by a multisynaptic pathway involving the lateral parabrachial nucleus and serotonergic neurons in the dorsal raphe nucleus. Glutamatergic neurons in the lateral parabrachial nucleus projects strongly to the dorsal raphe nucleus (Lee et al., 2003). Studies in rats have revealed that exposure to warm ambient temperature activates serotonergic neurons within the dorsal raphe nucleus, including those in the interfascicular part of the dorsal raphe nucleus (DRI) (Hale et al., 2011), which has been implicated in antidepressant-like behavioral effects (Lowry et al., 2007; Hale et al., 2013). Anatomical studies in rhesus monkeys and macaques have revealed that innervation of the medial orbitofrontal cortex arises specifically from the DRI region, that is, in the caudal part of the dorsal raphe nucleus, between the medial longitudinal fasciculi (Porrino and Goldman-Rakic, 1982; Cavada et al., 2000). Together with emerging evidence that serotonin functions in the orbitofrontal cortex to either facilitate exploitation of current resources or exploration of alternatives based on reward expectations (Roberts, 2011), and to coordinate positive affective responses (Man et al., 2012), these findings are consistent with a potential role of DRI serotonergic neurons projecting to the medial orbitofrontal cortex in positive affective responses to warm temperature. DRI neurons also appear to innervate the ventral striatum (Van Bockstaele et al., 1993) and anterior cingulate cortex (Porrino and Goldman-Rakic, 1982), regions that, together with the medial orbitofrontal cortex, show correlations with subjective pleasantness ratings made to warm and cold stimuli (Rolls et al., 2008). Overall, serotonergic systems may be involved in the effects of warm temperature on interpersonal warmth and prosocial behavior as evidence suggests that serotonin is an important modulator of social behavior, including attachment formation, social bonding, and social perceptions (Raleigh et al., 1980, 1991; Bilderbeck et al., 2011, 2014; Kiser et al., 2012; Ellingsen et al., 2014).

#### How does information about skin temperature reach brain regions involved in negative affective responses to cold temperature?

As with the medial orbitofrontal cortex, mentioned above, the neural pathways through which sensory information about cold temperature reaches the lateral orbitofrontal cortex, associated with unpleasant sensations, has not been defined. Thus, determining the relative contributions of the spinothalamic and spinoparabrachial pathways will require further study. A similar argument could be made that cold signals are relayed to the lateral orbitofrontal cortex by serotonergic neurons in the dorsal raphe nucleus. Studies in rats have revealed that exposure to cold temperature, similar to exposure to warm ambient temperature, activates serotonergic neurons within the dorsal raphe nucleus, including those in the DRI (Kelly et al., 2011). Presumably, warm sensitive and cold sensitive DRI serotonergic neurons are distinct, but this has not been determined empirically. Anatomical studies in macaques have revealed that innervation of the lateral orbitofrontal cortex also arises specifically from the DRI region, referred to as the dorsal raphe central superior nucleus in this study (Morecraft et al., 1992) (for a detailed discussion of the nomenclature of DRI serotonergic neurons, see Lowry et al., 2008a). These findings are consistent with a potential role of cold sensitive DRI serotonergic neurons projecting to the lateral orbitofrontal cortex in negative affective responses to cold temperature.

#### Making the clinical link: thermosensitive brain regions function abnormally in mood disorders

We turn now to evidence that the findings discussed thus far are of more than mere academic interest. Rather, many of the brain regions most implicated in registering—and reacting to—thermal signals from the periphery have been shown to function abnormally in patients with mood disorders (Table 1). For example, medial orbitofrontal cortex, as well as the pregenual cingulate cortex and the ventral striatum (areas associated with sensations of pleasant stimuli, thermal and otherwise), demonstrate decreased activity in depressed patients (Drevets et al., 2008). Conversely, the activity of more lateral regions of the orbitofrontal cortex (an area associated with sensations of unpleasant thermal stimuli), is increased in depressed patients (Drevets et al., 2008). On the other hand, activation of the medial orbitofrontal cortex and striatum, which is decreased in patients with unipolar depression, is increased in bipolar I disorder (Hulvershorn et al., 2012; Linke et al., 2012), suggesting underactivity and overactivity of regions involved in affective responses to pleasant, warm signals in unipolar and bipolar mania/hypomania, respectively. Conversely, activation of the lateral orbitofrontal cortex, associated with responses to cold temperature, which is increased in depressed patients, is decreased in bipolar patients in the hypomanic or manic phase compared to both the bipolar euthymic condition and healthy controls (Hulvershorn et al., 2012). Similar to these differences in activity in the orbitofrontal cortex in unipolar and bipolar disorders, the activity of the striatum (an area associated with sensations of pleasant stimuli) is decreased in depressed patients (Drevets et al., 2008; Hamilton et al., 2012), but increased in bipolar patients, in the euthymic, hypomanic or manic phases (Blumberg et al., 2003; Marchand and Yurgelun-Todd, 2010; Hulvershorn et al., 2012). The final region in which activation is correlated with subjective pleasantness ratings made to warm (41°C) and cold (12°C) stimuli, the pregenual anterior cingulate cortex, has been proposed to be involved in the shift from negative to positive affective bias following antidepressant treatment (Victor et al., 2013), consistent with microstimulation studies in primates showing that this region contains neurons representing motivationally positive and negative subjective value (Amemori and Graybiel, 2012), and consistent with recent findings that this region, and the adjacent dorsal anterior cingulate cortex, are associated with the anti-anhedonic effects of ketamine, a novel, rapid-acting antidepressant (Lally et al., 2014). Finally, evidence suggests that activity of the posterior insula, which mediates the discriminative aspects of thermosensation, is decreased in those at risk for unipolar depression in tasks associated with processing reward (McCabe et al., 2012; Sliz and Hayley, 2012), and increased in patients with bipolar disorder who are manic or hypomanic (Hulvershorn et al., 2012). Thus, the function of a network of brain regions implicated in subjective pleasantness and discriminative responses to warm and cold stimuli may be altered in unipolar and bipolar disorders, consistent with the hypothesis that dysregulation of thermosensory pathways and/or processing is associated with affective disorder.

**Table 1:** **Neural systems mediating affective responses to warm temperature are dysregulated in affective disorder**.

**Brain region**	**MDD^a^**	**Warmth^c^**	**BD^b^ (mania/hypomania)**	**Affective function**	**DRI projections**
Anterior cingulate cortex	↓	↑		Positive	Porrino and Goldman-Rakic, 1982
Medial orbitofrontal cortex	↓	↑	↑	Positive	Porrino and Goldman-Rakic, 1982; Cavada et al., 2000
Ventral striatum	↓	↑	↑	Positive	Van Bockstaele et al., 1993
Lateral orbitofrontal cortex	↑	↓	↓	Negative	Morecraft et al., 1992

a *Drevets et al., 2008*.

b *Hulvershorn et al., 2012; Linke et al., 2012*.

c *Rolls et al., 2008*.

It is not yet clear if the affective responses to warm temperature are mediated by the spinothalamic pathway, the spinoparabrachial pathway, or both, However, since warming-induced activation of the preoptic area in humans is correlated with activation of the insula and dorsal cingulate cortex (Farrell et al., 2014), and cutaneous warmth is relayed to the preoptic area by the spinoparabrachial pathway (Nakamura and Morrison, 2010), it is likely that the spinoparabrachial pathway plays an important role in relaying signals of affective warmth to cortical structures integrating positive affective responses.

## Evidence linking thermosensation with affective states relevant to well-being and depression

### Physical warmth promotes interpersonal warmth: psychological perspectives

Recent human research in embodied social cognition has explored the emotional and behavioral consequences of exposure to warm (vs. cold) objects and local ambient temperatures. This research examines the overlap between the processing of physical temperature information and any corresponding change in psychological states associated with temperature, such as metaphorical warmth or coldness (being a warm person Williams and Bargh, 2008), loneliness and social exclusion (Zhong and Leonardelli, 2008), and social closeness (Ijzerman and Semin, 2009, 2010). For example, in one study Williams and Bargh (2008) briefly exposed participants to either a hot or iced cup of coffee and, after a delay, asked participants to rate an ambiguous person on traits metaphorically associated with warmth (e.g., generosity, sociability) as well as traits unrelated to warmth (e.g., strength, honesty). The authors found that those exposed to the physically warm object saw more “warmth” in the ambiguously described person. Extending these findings, researchers have explored the downstream behavioral consequences of exposure to warm vs. cold temperatures. Exposure to warmth led people to prefer a gift for a friend over a reward for themselves (Williams and Bargh, 2008) and was associated with more trusting behavior in an economic game (Kang et al., 2011). In the consumption domain, researchers find that people prefer to watch romantic movies when they feel cold (Hong and Sun, 2012). Interestingly, the relationship between physical warmth and perceptions of social warmth appears to be bidirectional.

Just as physical warmth promotes perceptions of social warmth; social perceptions are capable of impacting perceptions of environmental temperatures. For example, Ijzerman and Semin (2009, 2010) found that just as exposure to ambient warm temperatures led people to feel socially closer to strangers, people reported feeling physically warmer when they thought about the similarities between themselves and others (prompting social closeness). In contrast, when people feel socially excluded, they feel physically colder, leading to lower estimates of ambient temperatures (Zhong and Leonardelli, 2008) and show decreased finger temperature (Ijzerman et al., 2012). Interestingly, social exclusion combined with exposure to a cold stimulus (cold tea cup) increases negative affect, whereas exposure to a warm stimulus (warm tea cup) alleviates the negative affect associated with social exclusion (Ijzerman et al., 2012).

Although such effects may initially seem surprising, we suggest that at least two perspectives that are not mutually exclusive make them comprehensible. We devote a good deal of discussion in this article to one of these perspectives, namely the likelihood that exposure to physically warm stimuli directly influences the activity of cortical structures involved in affective function. Here we touch upon a second possibility, and that is that these associations are to a large degree the result of the associative nature of memory (Tulving and Schacter, 1990). The idea here is that exposure to physically warm stimuli tends to activate the concept of “warmth” in working memory, which in turn activates schematically linked concepts, feelings, and action tendencies. When such information is active in working memory, it is likely to shape people's judgments, feelings, and behaviors.

If the mechanistic underpinnings of the effects of physical warmth on interpersonal interactions indeed involve the associative nature of memory, how does the mental association between physical and psychological warmth develop? Williams et al. (2009) theorized that such an association should result from early experiences with caregivers, in which exposure to a physically warm object (namely, another human) is repeatedly associated with emotional comfort and security. Young mammals crave close contact with their maternal caregiver (Harlow, 1958; Denenberg, 1968) and humans are no exception (Korner and Thoman, 1972; Brazelton, 1990). Indeed, such contact is critical for human social, emotional, and cognitive development (Bowlby, 1969; Ainsworth, 1979). As children develop the concepts of love, trust, and affiliation from their caregivers, they do so while being simultaneously exposed to warm caresses. One implication of this reasoning is that children who do not feel the physical warmth of their caregiver's touch should have a weaker association between physical and psychological warmth. Recent research bears out this prediction. Ijzerman et al. (2013) found that children exposed to physical warmth are later socially warmer, as demonstrated by activities such as sharing more stickers with their peers. However, this effect was only evident for children who were securely attached to their parents. The sharing behavior of insecurely attached children was not influenced by exposure to warmth cues (Ijzerman et al., 2013). Such findings support the view that the association between physical and psychological warmth in memory is the result of early life experiences.

Our understanding of the psychological effects of exposure to warm temperatures is bolstered by recent functional neuroimaging work examining the neural correlates of perceptions of physical and psychological warmth. Consistent with earlier studies, Inagaki and Eisenberger (2013) found that exposure to a physically warm pack (vs. a room temperature ball) led participants to report stronger feelings of social connection to close friends and family. Conversely, reading positive messages from close friends and family (vs. neutral messages) led participants to report feeling physically warmer. Using fMRI, these researchers observed that exposure to physical and social warmth cues independently activated overlapping regions of the middle insula and ventral striatum (see also Kang et al., 2011). These regions have been shown to be involved with temperature perception (Davis et al., 1998) and emotional processing (Craig, 2009). Findings such as these, while preliminary, begin to bridge the gap between the emotional and behavioral consequences tied to physical warmth perceptions (Williams and Bargh, 2008) and the underlying neural mechanisms through which such consequences arise, as discussed at length in this article. Moreover, it should be noted that brain-mediated associations between psychological and physiological warmth, while metaphorical, build upon actual physiological connections between physical warmth early in life and thermoregulation. For example, a recent study demonstrates that skin-to-skin contact during the first 24 h of postnatal life significantly reduces the development of hypothermia in newborns (Nimbalkar et al., 2014).

We have discussed that early life experiences cause people to form an association between physical and psychological warmth, and that this association is also expressed in common neural mechanisms for physical and psychological warmth (Inagaki and Eisenberger, 2013). It thus seems that an individual's attachment style, or at least having developed this association between physical and psychological warmth is very important when processing thermosensory information. The question that comes to mind is: what is the importance of having a(n) (in)secure attachment style (or associating physical warmth with psychological warmth) in relation to affective disorders. For example, are people with an insecure attachment style, and thus presumably a weaker association between physical and psychological warmth, more vulnerable to developing depression? Evidence is emerging to support an association between insecure attachment styles and vulnerability to depression. Insecure attachment style was found to be significantly related to rates of depression in a 12-month period (Bifulco et al., 2002a). Furthermore, studies using logistic regression analysis have shown that dysfunctional (“non-standard;” Enmeshed, Fearful, or Angry-dismissive style) attachment style (odds-ratio, 2.34; *p* < 0.02), poor support (odds-ratio, 2.09, *p* < 0.04), and childhood neglect/abuse (odds-ratio, 2.46, *p* < 0.01) provide the best model for predicting depression in a 12-month period (Bifulco et al., 2002b).

Although we have highlighted studies that support the possibility that complex bidirectional relationships exist between emotional warmth and physical warmth (i.e., warm temperature), it is important to note that not all studies have found these relationships. For example, Donnellan et al. (2012) have recently failed to replicate earlier findings that people use warm showers and baths to compensate for a lack of social warmth, and LeBel and Campbell (2013) have failed to confirm that individuals with high anxious attachment styles have heightened sensitivity to temperature cues. Importantly, both these negative replication studies included significantly larger subject samples than had the original positive reports. Nonetheless, other very recent studies have continued to find associations between emotional/cognitive state and temperature. For example Schilder et al. (2014) have replicated earlier findings that a simple cue of physical warmth makes people more likely to adopt a relational focus, and in a large sample Ijzerman et al. (2014) found that thinking positively about perceived communal commercial brands leads to heightened temperature estimates. We leave it to future research to more definitively bear out a full account of when and why warm temperatures promote warm feelings, and vice versa (e.g., by highlighting important moderators of these effects). Nonetheless, such embodied cognition findings are consistent with an independent stream of research showing that thermosensory experiences shape affective experience via changes in cortical activity.

### Interpersonal warmth promotes physical warmth

While the effects of physical warmth on interpersonal warmth have received considerable attention, the effects of interpersonal warmth on physical warmth (i.e., increased cutaneous temperature associated with increased cutaneous blood flow, and associated heat dissipation/thermoregulatory cooling) have received less attention. There is some evidence to support the hypothesis that interpersonal warmth increases cutaneous warmth, due to an increase in cutaneous blood flow, which is associated with thermoregulatory cooling. For example, one study found that brief social contact by the experimenter to intimate parts of the body (the face or chest) of an experimental subject, but not social contact to the outer arm or palm, induced increases in facial and chest temperature of the individual being touched, and these effects tended to be greater when the social contact was from an opposite-sex experimenter relative to a same-sex experimenter (Hahn et al., 2012). Another study found that facial temperature increases when an experimenter moves from social space to intimate space of the experimental subject; in addition, direct gaze, relative to averted gaze, increases facial temperatures of the subject and this effect is greater in the intimate space condition (Ioannou et al., 2014). It is unlikely that these responses are due to perceived threat, as negative emotions including fear (Kistler et al., 1998; Nakayama et al., 2005; Kuraoka and Nakamura, 2011), stress (Pavlidis et al., 2012), and guilt (Ioannou et al., 2013) result in a drop in the temperature of the nose, maxillary area, and forehead, as well as of the fingers due to peripheral vasoconstriction. In contrast, facial temperatures increase in association with sexual arousal in young men watching an erotic movie (Merla and Romani, 2007). Increased blood flow to the skin may in turn increase personal warmth, as slight increases in facial skin redness are perceived as more attractive (Stephen et al., 2009; Re et al., 2011), in effect completing a positive feedback loop wherein interpersonal warmth promotes physical warmth, and physical warmth promotes interpersonal warmth. It remains to be determined what aspects of interpersonal warmth result in increased cutaneous blood flow and associated thermoregulatory cooling, as well as the degree to which these effects are evident in different peripheral cutaneous vascular beds. Thermal infrared imaging promises to be useful for investigation of affective states and their physiological correlates in social situations.

### Discriminative, affective, and homeostatic components of thermosensation are altered in depressed patients

Individuals with affective disorders show altered perception of temperature, altered affective responses to changes in cutaneous temperature, as well as dysregulated thermoregulatory processes. Indeed, altered thermoregulatory cooling mechanisms among individuals with depression is a well-established clinical observation, leading some to the hypothesis that resting skin conductance levels, a measurement of sweat-induced moisture on the skin, may be a sensitive and specific marker for depression (Ward et al., 1983). The following sections review the evidence for altered discriminative, affective and thermoregulatory responses among individuals with affective disorders.

#### Discriminative aspects of thermosensation

Depressed patients show altered discriminative responses to heat, in the non-noxious (42°C) and noxious (44, 46°C) range (Strigo et al., 2008). In one study, unmedicated subjects with MDD rated heat stimuli as more intense, compared to healthy controls (Strigo et al., 2008). However, a number of studies have found the opposite, as measured by increased pain thresholds to noxious cold (Schwier et al., 2010) or heat (Bar et al., 2005, 2011) in depressed patients, relative to healthy controls. Further studies of discriminative responses to cold and heat in the non-noxious range are required to further explore potential differences in discriminative aspects of thermosensation in depressed patients and healthy controls.

#### Affective aspects of thermosensation

Depressed patients show altered affective responses to heat, both in the non-noxious (40, 42°C) and noxious (44, 46°C) ranges (Strigo et al., 2008). Subjects with MDD rate heat stimuli as more unpleasant, compared to healthy controls (Strigo et al., 2008). Perhaps more interestingly, depressed patients show a greater affective bias (unpleasantness rating/intensity rating) compared to non-depressed controls, particularly in the non-noxious heat range (Strigo et al., 2008), and show a lower affective threshold (i.e., the temperatures that are not unpleasant to the controls are highly unpleasant to the depressed subjects) even following happy and sad mood induction procedures (Ushinsky et al., 2013). These findings led the authors to propose the concept of “emotional allodynia” to explain the increased affective bias in MDD subjects, that is, a qualitatively altered negative emotional response to normally non-aversive thermal stimuli. The insensitivity of emotional allodynia to happy or sad mood induction procedures in MDD subjects suggests that this may be a chronic characteristic of MDD subjects. In these same subjects, differences in heat pain intensity thresholds were not different between MDD and control subjects, suggesting that these effects were not due to changes in nociceptive signaling *per se* (Ushinsky et al., 2013). Regardless, “emotional allodynia” would be expected to reduce the “window” for pleasant warmth, while increasing that for unpleasant heat.

#### Homeostatic aspects of thermoregulation

Although discriminative and affective aspects of thermosensation in the non-noxious range have received relatively little attention, far more information has been uncovered in relation to homeostatic dysregulation of thermoregulation in depressed patients. It is particularly well-established that depressed patients have dysfunction of thermoregulatory cooling mechanisms. It remains uncertain, however, if altered thermoregulatory cooling in MDD results from dysregulation of the afferent limb of thermal signaling, its integration, or efferent control of thermoregulatory cooling.

Regardless of the underlying mechanisms, depressed patients sweat less well-than healthy individuals. The observation that depressed patients have low skin conductance levels (an indirect measure of sweating), was first made in 1890 (Vigouroux, 1890). Since then, many studies have confirmed this original observation. In fact, so reliable is the association between decreased sweating and depression, that daytime resting skin conductance was proposed in the 1980s as *a potentially sensitive and specific marker for depression* (Ward et al., 1983). This suggestion was based on repeated findings that mean basal skin conductance levels are lower in unmedicated or medicated depressed subjects compared with controls and that low daytime resting skin conductance levels are highly predictive of MDD (McCarron, 1973; Dawson et al., 1977, 1985; Mirkin and Coppen, 1980; Carney et al., 1981; Donat and McCullough, 1983; Ward et al., 1983; Williams et al., 1985; Ward and Doerr, 1986).

These findings have been extended to patients with both unipolar and bipolar affective disorder (Iacono et al., 1983), panic patients with comorbid depression (Argyle, 1991), and non-clinically depressed individuals in a community sample (Kemp et al., 2005). In a recent study of children with a mean age of 8.7 years, low basal skin conductance levels were found to be a risk factor for depressive symptoms, as measured using the Children's Depression Inventory (CDI) (El-Sheikh and Arsiwalla, 2011). Specifically, decreases in sleep duration were associated with more depressive symptoms, but only for children with lower basal skin conductance levels. Similarly, when sleep efficiency was low, children with lower basal skin conductance levels had high rates of depression, relative to children with higher basal skin conductance levels.

A study by Nagai et al. (2004), has shown that neural activity in the ventromedial prefrontal cortex, a region associated with the “default mode” of brain function that is altered in depressed patients, as well as neural activity in the orbitofrontal cortex, covaries with skin conductance level, an index of sympathetic tone, during biofeedback and relaxation tasks, suggesting that low sympathetic tone, at least as indicated by decreased basal skin conductance levels, is integrally related to altered ventromedial and orbitofrontal prefrontal activity in depressed patients. As these investigators noted, these and other findings implicate the ventromedial and orbitofrontal prefrontal cortices in the generation of “somatic markers” that guide adaptive behavior. They suggest that, in the case of the ventromedial prefrontal cortex, associations with skin conductance reflect “a task-independent representation of background states of relaxation consistent with a proposed role in mediating a default baseline homeostatic state of brain activity.” On the other hand, associations between orbitofrontal cortex and skin conductance are better understood as a “mechanism mediating background hedonic aspects of feeling states linked to levels of corresponding bodily arousal and relaxation.” Highlighting the close association between thermoregulatory functioning and CNS abnormalities linked to depression is the fact that these brain areas have been repeatedly associated with MDD. However, it is important to note that these same areas have been implicated in many other non-thermosensory/regulatory functions as well.

In addition to low skin conductance levels at rest (electrodermal hypoactivity), a number of studies have documented reduced skin conductance responses to auditory stimuli (electrodermal hyporeactivity) in depressed patients, particularly patients with psychotic depression. Moreover, studies have found that electrodermal hyporeactivity is particularly associated with a history of suicide attempts (Edman et al., 1986; Thorell et al., 1987). Based on a meta-analysis, the sensitivity and specificity of electrodermal hyporeactivity for suicide in depressed patients were 97% and 93%, respectively (Thorell, 2009).

There may be both state and trait aspects of low skin conductance levels in depressed patients. Suggesting that decreased sweating may be a trait that increases vulnerability to MDD, studies reported that 1 and 2 years following recovery from a depressive episode subjects continued to demonstrate skin conductance levels in the low end of the normal range (Iacono et al., 1983, 1984). In agreement with these findings, two other studies found that skin conductance levels do not normalize in association with acute clinical recovery following treatment with antidepressants or electroconvulsive therapy (Dawson et al., 1977; Storrie et al., 1981). However, other studies suggest that decreased skin conductance, to at least some degree, reflects the depressive state itself. For example, one study found a correlation between increased basal skin conductance levels following 8 weeks of treatment with fluoxetine and percent improvement in Beck Depression Inventory (BDI) scores (Fraguas et al., 2007), while Bagg and Crookes found that palmar digital sweating increased in association with clinical recovery following electroconvulsive therapy (Bagg and Crookes, 1966). The latter findings are consistent with clinical studies reporting excessive sweating (an important thermoregulatory cooling mechanism in humans) as a common “side effect” of treatment with the majority of currently available antidepressant agents (e.g., tricyclic antidepressants, selective serotonin reuptake inhibitors, serotonin-norepinephrine reuptake inhibitors) (Marcy and Britton, 2005). Nevertheless, the issue of whether basal skin conductance levels increase following successful clinical outcomes requires further study, as does the intriguing possibility that increased sweating in response to antidepressants may either portend or correlate with clinical response.

It should be noted that other variables besides thermoregulation alter skin conductance. For example, the anticipation or performance of diverse tasks, including tasks requiring close attention to external stimuli or internal informational processing, increases skin conductance levels (Dawson et al., 2007). Consequently, it remains possible that low skin conductance levels in MDD are secondary to decreased engagement with internal or external tasks, rather than secondary to altered thermosensory/thermoregulatory systems.

Studies in young adults at genetic risk for affective disorder suggest that low basal skin conductance levels are not likely to be a genetic marker for affective disorder (Zahn et al., 1989). An alternate hypothesis is that low skin conductance levels, associated with increased risk of MDD, are a result of stressful experiences, either during early life, or adulthood, resulting in a shift from proactive to reactive emotional coping responses. Adverse life experiences result in a shift from proactive to reactive emotional coping styles in animal model systems. For example, maternal separation, or repeated exposure to psychosocial stress during adulthood, results in a shift from proactive to reactive emotional coping strategies (Gardner et al., 2005; Paul et al., 2011). The balance of proactive and reactive emotional coping is thought to be determined by the relative activity of the dorsal (proactive) and ventrolateral (reactive) parts of the periaqueductal gray, which are controlled by visceromotor systems that are dysregulated in depressed patients (Drevets et al., 2008). The ventrolateral periaqueductal gray, in particular, promotes reactive coping, characterized by behavioral quiescence, hyporeactivity to environmental stimuli, hypotension, bradycardia, and opioid-mediated analgesia (Lovick, 1993; Keay and Bandler, 2001). The periaqueductal gray, in turn, is controlled by visceromotor systems in the orbitofrontal and medial prefrontal cortices (Keay and Bandler, 2001). Individual variability along the spectrum of proactive vs. reactive emotional coping styles in response to stressors has been documented in diverse vertebrate and invertebrate species, including humans (Koolhaas et al., 1999). Operationally, a proactive coping response to a stressor, originally defined as the fight-flight response by Cannon (1915), is characterized behaviorally by territorial control and aggression. In contrast, a reactive coping response to a stressor, originally defined as a conservation-withdrawal response by Engel and Schmale (1972), is characterized behaviorally by immobility and low levels of aggression.

Physiological correlates of a reactive emotional coping style to stress are remarkably similar to those associated with depression, raising the possibility that a reactive emotional coping style shares some similarity to vulnerability to a depressive-like syndrome. Physiological characteristics of a reactive emotional coping style include high hypothalamic-pituitary-adrenal (HPA) axis reactivity, low sympathetic reactivity, high parasympathetic reactivity, and low testosterone (for review, see Koolhaas et al., 1999). Depression is associated with hypothalamic-pituitary-adrenal (HPA) axis hyperactivity (Pariante and Lightman, 2008), low sympathetic reactivity (at least as indicated by basal skin conductance levels and skin conductance response, as discussed above), high parasympathetic reactivity (Shinba, 2014), and low testosterone (Shores et al., 2005; Almeida et al., 2008; Hintikka et al., 2009; Giltay et al., 2012), but see (Araujo et al., 1998; Amiaz and Seidman, 2008; Berglund et al., 2011). It's clear that these physiological parameters do not apply to all depressed patients, however. For example, a subset of patients with co-morbid panic have extraordinarily high sympathetic activity, including high sympathetic outflow to the heart (Barton et al., 2007), and associations with higher blood pressure levels, higher incident hypertension, hypotension, and circadian variation alterations of these variables are described in depressed patients (Scalco et al., 2005).

#### Elevated body temperature in depression

Consistent with the hypothesis that depressed patients have dysfunction of thermoregulatory cooling mechanisms, a number of studies have found that individuals with affective disorders have elevated body temperature. Elevated body temperature at night, a time when thermoregulatory cooling responses are important for sleep onset and sleep quality (Raymann et al., 2005, 2008; Romeijn et al., 2011), is the most consistently observed circadian abnormality in depression (Avery et al., 1982b; Souetre et al., 1988; Duncan, 1996), an abnormality that typically normalizes with clinical improvement, (e.g., following ECT, antidepressant drug treatment or spontaneous recovery) (Avery et al., 1982a,b). Although not observed in all studies, a phase advance in the 24-h pattern of body temperature has also been described in some depressed patients (Dietzel et al., 1986; Goetze and Tolle, 1987; Souetre et al., 1988; Parry et al., 1989). Interestingly, despite the higher nocturnal temperature in depressed patients, there is no concomitant increase in nighttime sweating (Avery et al., 1999). Furthermore, several lines of evidence suggest that altered thermoregulatory cooling mechanisms are not specific to MDD. For example, an increase in nocturnal temperature is also observed in the depressed phase of bipolar disorder (Souetre et al., 1988) and seasonal affective disorder (SAD) (Schwartz et al., 1997b). As well as elevated night time temperature, individuals with MDD also have higher daytime (morning) temperature (Rausch et al., 2003) that is predictive of MDD. Patients with MDD have also been reported to demonstrate increased mean 24-h core body temperature and reduced circadian temperature amplitude when compared to normal controls (Szuba et al., 1997). Interestingly, when treated with ECT, both the absolute temperature and circadian temperature amplitude reverted to values observed in the control subjects. Finally, the short allele of the serotonin transporter linked polymorphic region (5-HTTLPR), which has been widely replicated as a risk factor for depression in response to psychosocial stress, has also been associated with increased oral body temperature in both depressed and non-depressed individuals (Rausch et al., 2003). These findings suggest that thermoregulatory cooling mechanisms are dysfunctional in depressed patients, consistent with the low basal skin conductance levels in depressed patients, but may be restored following clinical recovery. Moreover, genetic risk factors for MDD may promote hyperthermia independently of current depressive status, again pointing to the intimate relationship between thermoregulatory activity and biological mechanisms important to the pathogenesis of MDD.

#### Does symptom profile (melancholic vs. atypical) impact core body temperature in MDD?

As with all current psychiatric diagnostic categories, MDD is not a homogeneous disease state, either in terms of etiology or phenomenology. Nor have phenomenological differences—such as the distinction between melancholic and atypical depression—mapped in any consistent way with differences in pathophysiology, although, of all phenomenological distinctions, the one between melancholic and atypical symptom patterns has been most studied. Given this, it is intriguing that we could find no study that directly compared core body temperature between individuals with melancholic vs. atypical symptoms. Nor could we find any studies that examined core body temperature specifically in atypical depression. Nevertheless, in a study of light therapy in women with borderline personality disorder, although general depression scores and borderline personality disorder symptoms were not affected, decreases in atypical depression scores following light therapy were associated with increased skin temperature during sleep, a measure that was interpreted as increased relaxation (Bromundt et al., 2013). This finding is consistent with the hypothesis that alleviation of atypical depression symptoms is functionally associated with increases in thermoregulatory cooling and elevation of cutaneous temperature.

A small, but fairly consistent, literature exists examining core body temperature in two mood disorders strongly associated with atypical symptoms and most common in young women: SAD and late luteal phase (formerly premenstrual dysphoric) disorder. Interestingly, as with melancholia, both conditions have been associated with increased core body temperature. Women with late luteal phase disorder demonstrate increased core body temperature when compared to women without the condition (Severino et al., 1991; Parry et al., 1997; Shechter et al., 2012). Although less data are available directly comparing core body temperature during winter seasonal depression and normal controls, it nonetheless appears that winter depression is associated with elevated body temperature. The data for SAD are particularly interesting because they demonstrate that successful treatment with light therapy reduces temperature and, in one study at least, that reduced body temperature was associated with improvement in depressive symptoms (Schwartz et al., 1997a). Interestingly, individuals have lower temperatures when euthymic in summer than when suffering from atypical depression in winter (Levendosky et al., 1991). This raises the intriguing possibility that the heat of summer might sensitize afferent thermosensory/regulatory pathways in much the same manner as WBH and that this might account to some degree for the improvement in symptoms seen in summer in this condition. This notion is a straightforward prediction of the theoretical framework espoused in this article, but to our knowledge has never been previously advanced. Finally, several small studies suggest that individuals with SAD have impaired ability to activate thermoregulatory cooling mechanisms (Arbisi et al., 1989, 1994), which, again, is remarkably consistent with the idea that skin-to-brain-to-skin thermosensory/regulatory circuitry is abnormal in depression, and likely across a range of depressive symptom subtypes.

Interestingly, increased self-soothing behaviors are characteristic of atypical depression. Scores on two self-comforting items (craving “comfort” foods, such as chocolate, and “warming up,” such as having a hot bath) increase linearly with the number of DSM-IV accessory atypical depressive symptoms (Parker and Crawford, 2007). These findings raise the question, do individuals with atypical depression engage in these self-soothing behaviors to (1) counteract low skin temperature or social coldness (i.e., feelings of having low resources), or (2) to trigger cooling mechanisms that downregulate sympathetic and emotional arousal as well as core body temperature? Both may be the case. Social exclusion leads to lower skin temperatures, and holding a warm beverage can alleviate negative affect experienced after social exclusion (Ijzerman et al., 2012). Thus, warm baths, by elevating cutaneous temperature, may alleviate feelings of social coldness or social exclusion and the associated negative affective states. In the study by Parker and Crawford (2007) the chocolate cravers group overall was found to have higher scores on the irritability, rejection sensitivity, anxious worrying, and self-focused scales, all derived from the higher-order neuroticism construct; irritability and rejection sensitivity were identified as predictors of chocolate craving status. As neuroticism has been defined in terms of limbic activation, with individuals with high scores in neuroticism characterized by intense autonomic discharges (Eysenck, 1967), these self-soothing mechanisms may trigger mechanisms that downregulate sympathetic and emotional arousal as well as core body temperature. Consistent with this hypothesis, of those that craved chocolate, the majority who rated chocolate's capacity to improve their mood as moderately or very important were more likely to indicate it made them feel less anxious and less irritated.

## Putting embodied perspectives to work: exploring the antidepressant potential of ascending thermosensory pathways

### Overview

With few exceptions, extant somatic treatments for depression (e.g., antidepressant medications, ECT) share in common mechanisms of action that simultaneously impact multiple areas of brain functioning, only a portion of which are likely to confer antidepressant benefit (Hale et al., 2013). Indeed, it is increasingly recognized that the current pharmacologic approach of targeting receptors and/or reuptake sites for neurotransmitter systems that impact widely distributed, and functionally divergent, brain regions may be approaching the limit of its effectiveness (Insel and Sahakian, 2012). One reason for our inability to materially enhance efficacy with this approach may be because it does not take full advantage of the highly complex functional topology of the CNS, and the corollary truth that the same neurotransmitter and/or receptor can have very different, and not infrequently opposing, physiological and behavioral effects depending on the anatomical pathway in which they are operating (Hale et al., 2012).

What is needed, therefore, is a methodology for enhanced anatomical (and hence functional) specificity in our interventions. This insight fuels current enthusiasm for both deep brain stimulation and transcranial magnetic stimulation (DBS and rTMS, respectively). Findings discussed below regarding the anatomical specificity of peripheral thermosensory pathway—CNS interconnectivity point to a different, but complementary, approach. Rather than attempting to alter neurocircuit functionality by technological methodologies (i.e., DBS, rTMS), it may be possible to harness anatomic/functional specificity built into peripheral sensory signaling pathways by evolutionary processes to selectively impact functioning in specific brain regions linked in animal and human studies to depression and well-being.

Supporting this approach, prior studies suggest that altering functional activity in such peripheral sensory pathways—in this case the innate immune inflammatory response—produces antidepressant effects without incurring some of the disadvantages inherent in directly manipulating the CNS with either drugs or devices. For example, utilizing a randomized, placebo-controlled design in 60 patients with treatment-resistant depression, we found that peripheral blockade of the proinflammatory cytokine tumor necrosis factor (TNF)-alpha with a biologic agent too large to cross the blood-brain-barrier (infliximab) produces antidepressant effects similar in magnitude to those seen with standard antidepressants in individuals with elevated baseline concentrations of peripheral inflammatory biomarkers [i.e., C-reactive protein (CRP) and TNF-alpha], while having no effect, or a detrimental effect compared to placebo, in patients with progressively lower baseline levels of these same biomarkers (Raison et al., 2013). Importantly, treatment with infliximab was associated with an incidence of side effects no greater than placebo, consistent with the notion that modulating peripheral pathways that signal to the CNS via specific, evolutionarily conserved, mechanisms induces antidepressant effects that are apparent primarily in those individuals with evidence of abnormal functioning in these same pathways, whilst largely avoiding adverse events incurred by the widespread alteration of neurotransmitter systems typically induced by antidepressants. It is to this possibility that we now turn, focusing on the use of WBH to sensitize (enhance activity) in one such peripheral signaling system, the ascending pathways that signal non-noxious warm stimuli from the periphery to the CNS.

### Evidence that ascending thermosensory pathways signal to serotonergic systems and brain areas implicated in MDD

**Figure 2:**
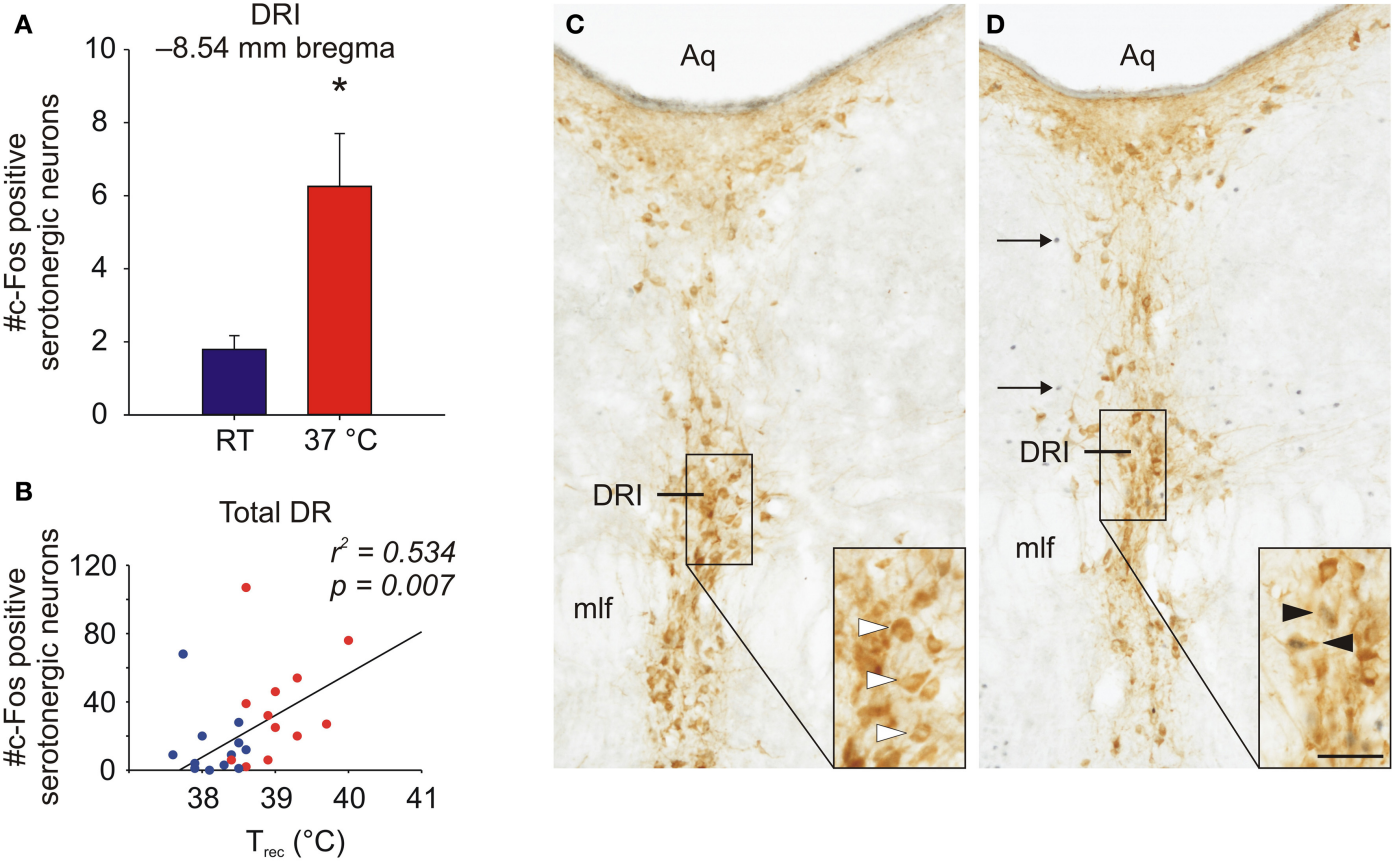
**Graphs illustrating effects of exposure of rats to warm ambient temperature on serotonergic neurons in the interfascicular part of the dorsal raphe nucleus (DRI)**. Rats were exposed to either room temperature (RT; 23°C) or (37°C) for 105 min. **(A)** Exposure to warm ambient temperature, relative to RT control conditions, activated DRI serotonergic neurons, as measured by double immunohistochemical staining of c-Fos, the protein product of the immediate-early gene, *c-fos*. **(B)** Scatter plot depicting the relationship between the post-whole body heating rectal temperature (Trec) of individual rats and the number of c-Fos-immunoreactive (ir) serotonergic neurons for all sampled subdivisions of the dorsal raphe nucleus (total DR). **(C,D)** Photomicrographs illustrating c-Fos and tryptophan hydroxylase (TPH) immunostaining in the dorsal raphe nucleus. Panels illustrate the DRI at −8.54 mm bregma of a rat exposed to **(C)** room temperature (RT) or **(D)** 37°C ambient temperature for 105 min. Black boxes in **(C)** and **(D)** indicate regions shown at higher magnification in insets in the lower right corner of each panel. White arrowheads indicate c-Fos-immunonegative/TPH-immunoreactive (ir) neurons, black arrowheads indicate c-Fos-ir/TPH-ir neurons and black arrows indicate c-Fos-ir/TPH-immunonegative cells. Abbreviations: Aq, cerebral aqueduct; mlf, medial longitudinal fasciculus. Scale bar, 100 μm **(A,B)**, and 50 μm, insets. Adapted from Hale et al. (2011), with permission.

Recent studies in rodents have demonstrated that non-noxious afferent warm or cold temperature signals activate brainstem serotonergic systems within the dorsal raphe nucleus (Hale et al., 2011; Kelly et al., 2011) (Figure 2), which are the major sources of serotonergic projections for forebrain limbic structures. Increasing evidence suggests that activation of brainstem serotonergic systems by warm temperature may be mediated by activation of the warm-sensitive spinoparabrachial pathway, given that the lateral parabrachial nucleus gives rise to dense projections to the dorsal raphe nucleus (Lee et al., 2003), and temperature dependent activation of the lateral parabrachial nucleus (LPB) is highly correlated with temperature dependent activation of serotonergic neurons within the dorsal raphe nucleus (Kelly et al., 2011). Based on these projections to the dorsal raphe nucleus, warm afferent thermal signals are subsequently relayed to forebrain structures implicated in the affective component of thermal responses, e.g., medial orbitofrontal cortex, pregenual cingulate cortex and the ventral striatum (Figure 1). In addition to multisynaptic thermosensory pathways involving the dorsal raphe nucleus, thermal signals may also reach these cortical areas via either spinothalamic pathways or via spinoparabrachial pathways that project directly from the LPB to forebrain systems (i.e., thereby bypassing the dorsal raphe nucleus).

Relevant to pathways that pass through the dorsal raphe nucleus, as discussed above the medial orbitofrontal and anterior cingulate cortices in primates are heavily innervated by neurons located in the interfascicular part of the dorsal raphe nucleus (DRI) (Porrino and Goldman-Rakic, 1982), a subset of serotonergic neurons that has been implicated in antidepressant-like behavioral effects in rodents (Lowry et al., 2007). In keeping with the potential for DRI serotonergic neurons to relay thermal signals to forebrain structures involved in affective responses to thermal signals, they are activated by exposure to either warm (Hale et al., 2011) or cold (Kelly et al., 2011) stimuli.

Although the reason why depression is associated with higher body temperature is not conclusively known, one of the strengths of the model presented here is that it provides a parsimonious explanation for this phenomenon. Specifically, if, as we posit, depression is associated (at least in some instances) with reduced signaling in the just-described afferent thermoregulatory pathway that mediates heat defense by inducing thermoregulatory cooling to maintain thermal homeostasis, it is clear that sub-optimal activity of this pathway would likely be associated with increased core body temperature as a result of impaired thermoregulatory cooling. Said more simply, elevated body temperature in depression reflects sub-optimal thermoregulatory cooling capacity. Indeed, because evidence suggests that the spinoparabrachial pathway that runs to thermoregulatory areas of the dorsal raphe nucleus also activates higher brain areas associated with positive emotional states, decreased activity in this pathway should produce both impaired thermoregulatory cooling (hence increased body temperature) and depressed mood. Conversely, stimulating this pathway should reduce body temperature and improve mood. Because WBH appears to activate this pathway, one would predict that it should have antidepressant effects and lower body temperature. We turn now to evidence that this is the case.

### Antidepressant properties of themosensory stimulation: evidence from whole-body hyperthermia (WBH)

#### Animal studies

We have shown in rodent models that WBH activates serotonergic neurons in the midbrain [DRI and dorsal raphe nucleus, ventrolateral part (DRVL)] that are implicated in both thermoregulatory cooling and antidepressant and anti-anxiety behavioral effects (Hale et al., 2011), while avoiding activation of other subregions of the dorsal raphe nucleus, including the dorsal part of the dorsal raphe nucleus (DRD), which has been implicated in the facilitation of anxiety states (Lowry et al., 2005, 2008b; Lowry and Hale, 2010; Rozeske et al., 2011). In addition, WBH decreased c-Fos expression in the raphe pallidus nucleus (RPa), consistent with activation of thermoregulatory cooling mechanisms (the RPa normally facilitates thermogenesis and vasoconstriction in cutaneous vascular beds and promotes sympathetic tone) (Hale et al., 2011). These findings are consistent with earlier animal work from our group showing that another peripheral intervention with antidepressant effects in the forced swim test (s.c., injection of the non-pathogenic, saprophytic mycobacterium, *Mycobacterium vaccae*) also activated DRI serotonergic neurons and resulted in increased serotonergic activity in cortical areas repeatedly implicated in the pathogenesis of MDD (Lowry et al., 2007).

#### Whole-body hyperthermia (WBH) demonstrates antidepressant effects in humans

Based on the theoretical considerations and animal data discussed above, one might predict that WBH in humans would induce two testable outcomes: a reduction in depressive symptoms and a lowering of core body temperature. In addition, if WBH works—at least in part—via activation and/or sensitization of the circuit that runs from peripheral biosensors in the skin and other bodily tissues to the brain areas discussed above (via ascending thermosensory pathways) and then back to the periphery (via descending thermoregulatory cooling pathways) one might predict that depressed subjects with evidence of abnormalities in this skin-to-brain-to-skin (S2B2S) circuit might be more likely than other depressed individuals to respond clinically to WBH. Although multiple readouts of S2B2S are likely to exist based on its functional anatomy (i.e., changes in autonomic tone, sleep, immune activity), the most direct measure of its activity is likely body temperature. People with sub-optimal S2B2S functioning would be expected to have impairments in thermoregulatory cooling and therefore to show elevations in core body temperature. Thus, following this line of reasoning, increased body temperature at baseline, potentially indicating dysregulation of thermoregulatory function, should—somewhat paradoxically—be associated with enhanced antidepressant responses to hyperthermia.

**Figure 3:**
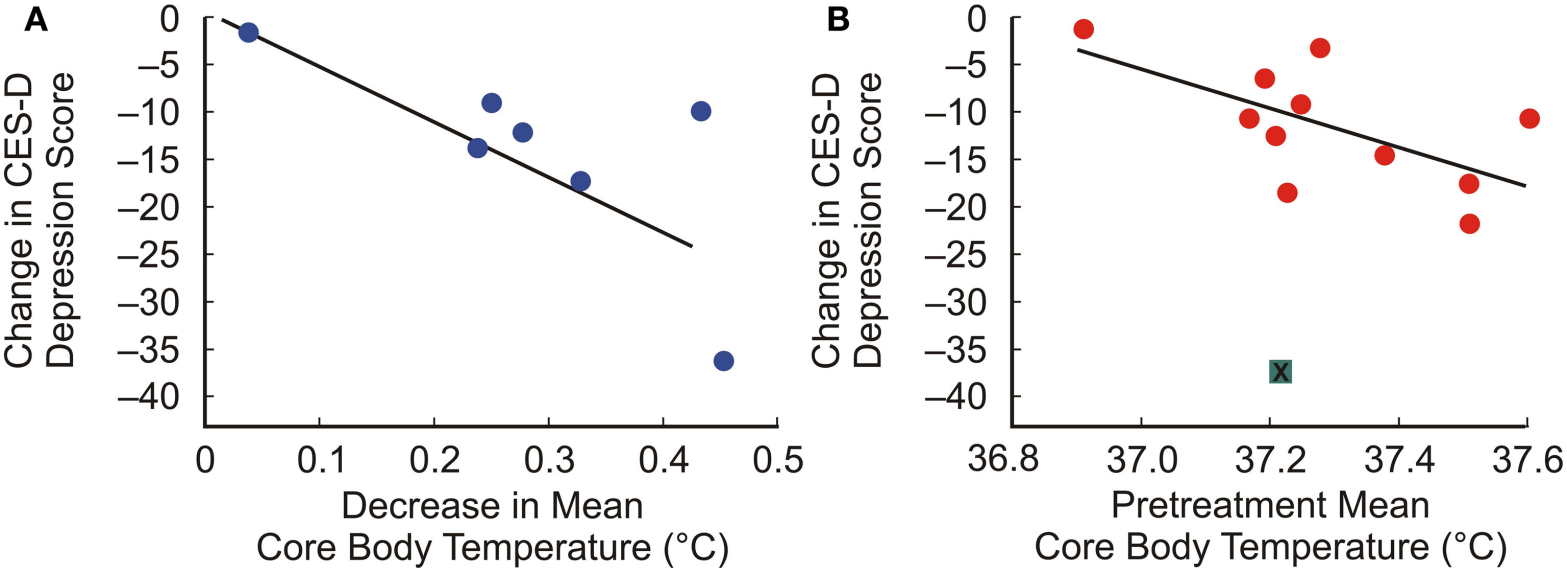
**Associations between 24-h mean core body temperature and depressive symptom response to whole-body hyperthermia**. **(A)** Scatterplot illustrating the correlation between decreases in score on the Center for Epidemiologic Studies Depression Scale (CES-D) and decreases in 24-h mean core body temperature from before treatment to 5 days after treatment with whole-body hyperthermia. **(B)** Scatterplot illustrating the correlation between 24-h mean core body temperature before treatment and changes in CES-D depression scores from before treatment to 5 days after treatment, with one outlier removed (indicated by a green box with an “x” through it). Adapted from Hanusch et al. (2013), with permission.

As an initial first test of these predictions we have conducted a small open trial examining the acute antidepressant effects of a single session of WBH (Hanusch et al., 2013). In 16 medically healthy adults with MDD, we found that WBH induces a rapid, robust and sustained reduction in depressive symptom scores [assessed with the German version of the Centers for Epidemiologic Studies Depression Scale (CES-D)] (Orme et al., 1986), based on CES-D scores dropping from 29.9 (*SD* = 10.6) pre-treatment to 19.2 (*SD* = 12.3) 5 days post-treatment [*t*_(15)_ = 4.53 *p* < 0.001, Cohen's *d* effect size = 1.13] (Hanusch et al., 2013). Thirteen of these subjects received no other pharmacologic or psychotherapeutic intervention during this period, whereas three subjects were on chronic treatment with a selective serotonin reuptake inhibitor (SSRI) antidepressant (with no change in dosage during the study period). Interestingly, when looked at separately, WBH appeared to have no effect in these three, and, in fact, worsened depressive symptoms in one of them. With the three subjects receiving SSRIs removed from analysis, the effect size of WBH increased [*t*_(12)_ = 5.15 *p* < 0.001, effect size = 1.4]. Interestingly, the antidepressant response observed 5 days post-treatment was actually improved 6 weeks later in nine patients for whom these longer-term follow-up data were available (CES-D mean score 13.9). Although these patients received additional treatment during this period, these findings, while preliminary, suggest that WBH may induce a rapid antidepressant response that can be solidified by longer-term modalities. We were able to obtain 24-h mean core body temperature at baseline and post-treatment day 5 for 7 of our patients who received WBH. A single session of WBH significantly reduced mean circadian core body temperature 5 days post-treatment [pre-treatment 37.3 (*SD* = 0.24); post-treatment 37.0 (*SD* = 0.14), *t*_(6)_ = 5.5, *p* = 0.002, effect size = 2.1], suggesting that WBH enhanced thermoregulatory cooling capability. Moreover, consistent with the possibility that the S2B2S circuit represents a functionally unified pathway, reductions in core body temperature from pre-treatment to post-treatment day 5 showed a large effect size correlation with reductions in depressive symptoms over the same period [*r*_(4)_ = 0.73, *p* = 0.06] (Figure 3). Finally as predicted, increased circadian mean core body temperature prior to treatment showed a large effect size correlation with improved CES-D depressive symptom responses 5 days following a single WBH session, [*r*_(9)_ = 0.62, *p* = 0.043] (Figure 3). Although this is the first study, to our knowledge, to examine WBH specifically for MDD, our findings are consistent with reports that WBH improves mood in patients with cancer and improves quality of life scores in patients with type II diabetes (Koltyn et al., 1992; Beever, 2010).

#### Convergence of cortical integration of affective warmth and affective touch

Recent work by Olausson and colleagues (Loken et al., 2009; McGlone et al., 2014) has led to significant advances in understanding how another stimulus associated with intimate affiliative behavior, that is gentle stroking or low-velocity/force stroking stimuli associated with affective touch, is signaled to the spinal cord and higher integrative centers in the CNS. There are many similarities to the neural systems mediating affective responses to pleasant tender, caress-like skin stimulation, and the neural systems mediating affective responses to warm temperature. Affective responses to pleasant touch are mediated by unmyelinated C low-threshold mechanoreceptors (CLTMs), referred to as C-tactile afferents (CTs) in humans. Early studies demonstrated that CTs may respond to cooling but not to warming or noxious heating (Nordin, 1990). Subsequently, it has been shown that CTs are tuned to tactile stimuli at normal skin temperatures (Ackerley et al., 2014); in other words, CT firing frequencies were greater in response to stroking of the skin when the probe was at 32°C, relative to when the probe was either cool (18°C) or warm (42°C). Moreover, pleasantness ratings were higher at neutral relative to either cool or warm temperatures, and a correlation between CT firing rates and pleasantness ratings was only observed at the neutral temperature. Consequently, affective touch and affective warmth seem to operate independently at the level of the primary sensory fibers. These data have been interpreted to support the mechanistic role of CTs in signaling pleasant skin-to-skin contact, promoting interpersonal touch and affiliative behavior.

There does seem to be convergence, however, of affective touch and affective warmth signals at the level of cortical structures integrating responses to affective stimuli. Like warm sensitive afferents, touch sensitive afferents have both discriminative functions and affective functions. Touch sensitive afferents relaying discriminative information are myelinated Aβ fibers enabling fast conduction velocities and rapid central processing. These signals are thought to be integrated first at the level of the dorsal horn of the spinal cord; they are then relayed via the spinothalamic pathways to the ventral thalamus, and finally the somatosensory cortex (SI/SII) for discriminative processing. Consistent with the dichotomy between discriminative and affective processing of warm temperature, distinct pathways mediate the discriminative and affective responses to pleasant touch. CLTMs project to inner lamina II of the spinal dorsal horn (Sugiura, 1996), which relay the signal to projection neurons in lamina I. As with warm sensitive afferents, low-velocity touch sensitive neurons in lamina I of the spinal dorsal horn project to the contralateral parabrachial nucleus (Andrew, 2010). Cortical processing of pleasant touch involves many of the same structures implicated in the processing of affective warmth, including the posterior insular cortex, pregenual anterior cingulate cortex (Lindgren et al., 2012), and midanterior orbitofrontal cortex (for review, see McGlone et al., 2014). In addition, top-down cognitive modulation of the affective representation of touch is associated with activity in the pregenual cingulate cortex, orbitofrontal cortex, and ventral striatum (McCabe et al., 2008; Rolls, 2010). The dichotomy between discriminative and affective responses to touch may not be absolute, as the perceived affective quality of a caress (perceived to be given by a male or female to heterosexual males) appears to involve the primary somatosensory cortices (Gazzola et al., 2012). Nevertheless, although pleasant touch and pleasant warmth have unique primary afferent sensory mechanisms, there is a high level of convergence of these signals in cortical structures integrating positive affective responses.

#### Convergence of cortical responses to affective warmth and rapid acting antidepressant therapy

Recent studies show that ketamine, a rapid acting antidepressant drug currently in development for treatment of affective disorders, activates the same positive affective circuits that we have highlighted here for WBH. That is, the medial orbitofrontal cortex, ventral striatum, and the dorsal anterior cingulate cortex, extending into the pregenual cingulate/callosal region (Lally et al., 2014). Interestingly, activations of the medial orbitofrontal cortex and ventral striatum were associated with improvements of overall depression scores as measured by the MADRS scores, while activation of the dorsal anterior cingulate cortex, extending into the pregenual cingulate/callosal region was associated specifically with the anti-anhedonia effects of ketamine, as measured using the Snaith-Hamilton pleasure scale (SHAPS), The SHAPS is a validated 14-item, self-administered scale that measures levels of anticipatory anhedonia (Leventhal et al., 2006; Franken et al., 2007; Nakonezny et al., 2010). Interestingly, the ability of ketamine to induce thermoregulatory cooling in animals (Fahim et al., 1973; Pietersen et al., 2006), resulting in reduced core body temperature, was a primary consideration in selection of ketamine for initial clinical studies for the off-label use of ketamine for treatment of children with pediatric bipolar disorder/fear of harm phenotype (Papolos et al., 2013), who appear to suffer from an inability to dissipate heat (Papolos et al., 2009). These findings suggest that WBH and ketamine, which both have strong thermoregulatory cooling effects, may activate the same positive affective or anti-anhedonic circuits, and that activation of these circuits, particularly the dorsal anterior cingulate cortex/pregenual cingulate cortex, may play a role in the anti-anhedonic effects.

## Conclusions

Across different levels of analysis (neuroanatomy, neurotransmission, and higher-order cognition), evidence converges on the perspective that thermosensory and thermoregulatory processes meaningfully contribute to the affective experiences of humans and non-humans alike. The extant literature points to bidirectional relationships between experiences with physical temperatures and affect, in both clinical and non-clinical populations. How important these thermosensory and thermoregulatory relationships will turn out to be in comparison to other biological and social factors that contribute to well-being and mental disorders remains an open question, the answer to which will ultimately determine the clinical utility of much of what has been discussed in this article. Based on preliminary findings from our group that a single session of WBH induced a rapid, large and sustained reduction in depressive symptoms in adults with major depression (Hanusch et al., 2013), we might suggest that thermosensory/thermoregulatory pathways hold therapeutic promise at least equal to other therapeutic approaches currently used in the treatment of MDD. If confirmed in ongoing randomized placebo-controlled trials, an important follow-up question will be whether thermosensory/thermoregulatory mechanisms contribute to depression in a widespread manner or exert their effects—both positive and negative—only in a subset of depressed individuals.

While available data are agnostic on this question, the ideas advanced in this paper suggest several clear hypotheses that will be amenable to testing in future research. First, and foremost, thermosensory pathways that convey non-noxious warm temperature signals to the brain should have antidepressant properties when stimulated. Preliminary work with WBH provides support for this prediction (Hanusch et al., 2013), but additional, randomized, placebo controlled trials will be required for confirmation that the effect is specific and of clear therapeutic relevance. Stimulation of at least one warm-sensitive ion channel, transient receptor potential cation channel, subfamily V, member 3 (Trpv3) induces anxiolytic and antidepressant-like behavioral responses in rodent models (Moussaieff et al., 2008). To our knowledge, no clinical trials have evaluated potential antidepressant effects of pharmacological activation of warm sensitive afferent pathways.

Second, at least a subset of depressed patients should show reduced functioning in pathways running from the skin to the brain and back to the skin that are essential for thermosensation and thermoregulation. We have reviewed evidence supporting this idea, for example data that depressed individuals sweat less well than others, but it remains unclear whether people with depression suffer primarily from abnormal functioning in CNS components of thermosensory/regulatory circuitry or whether insufficient afferent signaling from the periphery might be a primary cause of depression in some individuals. The very fact that heating the body appears to reduce depressive symptoms suggests that the brains of responsive individuals may not harbor any type of primary pathology that make them unresponsive to a “jolt” from the periphery. Indeed an intriguing third hypothesis arising from the ideas articulated in this paper is that some individuals with MDD may have primary abnormalities not in the CNS but in peripheral signaling tissue. For example, some people with MDD might suffer because of insufficient innervation or functionality of skin-embedded biosensors. Specifically stimulating these biosensors might produce an antidepressant effect without requiring that the modality involved induces persistent functional adaptations in CNS functioning whatsoever. Regardless of the level of impaired functioning within skin-to-brain-to-skin thermosensory/regulatory systems, a clear prediction of the model espoused here is that elevations in core body temperature that have been observed in MDD should be associated with reduced capacity to activate thermoregulatory cooling, as indexed by phenomena such as reduced cutaneous vasodilation or reduced sweating capacity. Assuming that insufficient activity in afferent thermosensory/regulatory pathways contributes to at least some cases of depression, a fourth hypothesis is that because manifestations of this reduced functioning would include increased body temperature and decreased sweating, one would predict that depressed individuals with increased core body temperature and/or decreased ability to sweat would preferentially respond to WBH. Again our preliminary data suggest that this might be the case (Hanusch et al., 2013).

Third, it is well-known that serotonergic antidepressants (which presumably stimulate—albeit indiscriminately—serotonergic signaling by thermoregulatory raphe neurons) increase one's propensity to sweat. A novel hypothesis derived from the ideas in this article is that increased sweating in response to these antidepressants should be associated with a degree of clinical improvement, because sweating is a potential read-out for activity in afferent thermosensory/regulatory pathways.

A final—and perhaps most radical—prediction is that people who respond best to a peripheral intervention like WBH might be found to have less architectural pathology within the CNS than those who don't respond and who might require a more CNS-specific type of intervention. Said differently, those most likely to benefit from stimulation of peripheral afferent thermosensory/regulatory pathways may have depression with a causal locus more completely located outside the CNS than those who don't respond. Nonetheless, these possibilities need to be balanced against the high likelihood that in many cases interactions between stimuli, sensory process, and changes in CNS function will turn out to be as relevant to pathogenesis as abnormalities isolated to any particular element in the skin-to-brain-to-skin thermosensory/regulatory circuit described in this article. As appears to be the case more generally, depressive pathology may well be distributed across the functioning of thermosensory/regulatory systems and may result to a significant degree from poor functioning of the system in its entirety.

However future research weighs in on these hypotheses and predictions, at the least it seems safe to say at this point that, given the importance and ubiquity of thermal sensations for all of human experience, further inquiry into the relationship between thermosensation, serotonergic neural activity, thermoregulation, and emotional well-being is warranted.

### Conflict of interest statement

The authors declare that the research was conducted in the absence of any commercial or financial relationships that could be construed as a potential conflict of interest.

## References

[B1] Ackerley R. Backlund W. H. Liljencrantz J. Olausson H. Johnson R. D. Wessberg J. (2014). Human C-tactile afferents are tuned to the temperature of a skin-stroking caress. J. Neurosci. 34, 2879–2883. 10.1523/JNEUROSCI.2847-13.2014 24553929 PMC3931502

[B2] Ainsworth M. D. (1979). Infant–mother attachment. Am. Psychol. 34, 932–937. 10.1037/0003-066X.34.10.932 517843

[B3] Almeida O. P. Yeap B. B. Hankey G. J. Jamrozik K. Flicker L. (2008). Low free testosterone concentration as a potentially treatable cause of depressive symptoms in older men. Arch. Gen. Psychiatry 65, 283–289. 10.1001/archgenpsychiatry.2007.33 18316674

[B4] Amemori K. Graybiel A. M. (2012). Localized microstimulation of primate pregenual cingulate cortex induces negative decision-making. Nat. Neurosci. 15, 776–785. 10.1038/nn.3088 22484571 PMC3369110

[B5] Amiaz R. Seidman S. N. (2008). Testosterone and depression in men. Curr. Opin. Endocrinol. Diabetes Obes. 15, 278–283. 10.1097/MED.0b013e3282fc27eb 18438177

[B6] Andrew D. (2010). Quantitative characterization of low-threshold mechanoreceptor inputs to lamina I spinoparabrachial neurons in the rat. J. Physiol. 588, 117–124. 10.1113/jphysiol.2009.181511 19933757 PMC2821553

[B7] Andrew D. Craig A. D. (2001). Spinothalamic lamina I neurones selectively responsive to cutaneous warming in cats. J. Physiol. 537, 489–495. 10.1111/j.1469-7793.2001.00489.x 11731580 PMC2278968

[B8] Araujo A. B. Durante R. Feldman H. A. Goldstein I. McKinlay J. B. (1998). The relationship between depressive symptoms and male erectile dysfunction: cross-sectional results from the Massachusetts Male Aging Study. Psychosom. Med. 60, 458–465. 10.1097/00006842-199807000-00011 9710291

[B9] Arbisi P. A. Depue R. A. Krauss S. Spoont M. R. Leon A. Ainsworth B.. (1994). Heat-loss response to a thermal challenge in seasonal affective disorder. Psychiatry Res. 52, 199–214. 10.1016/0165-1781(94)90088-4 7972575

[B10] Arbisi P. A. Depue R. A. Spoont M. R. Leon A. Ainsworth B. (1989). Thermoregulatory response to thermal challenge in seasonal affective disorder: a preliminary report. Psychiatry Res. 28, 323–334. 10.1016/0165-1781(89)90213-8 2762433

[B11] Argyle N. (1991). Skin conductance levels in panic disorder and depression. J. Nerv. Ment. Dis. 179, 563–566. 10.1097/00005053-199109000-00008 1919559

[B12] Atlas L. Y. Lindquist M. A. Bolger N. Wager T. D. (2014). Brain mediators of the effects of noxious heat on pain. Pain 155, 1632–1648. 10.1016/j.pain.2014.05.015 24845572 PMC4104234

[B13] Augustine J. R. (1996). Circuitry and functional aspects of the insular lobe in primates including humans. Brain Res. Brain Res. Rev. 22, 229–244. 10.1016/S0165-0173(96)00011-2 8957561

[B14] Avery D. H. Shah S. H. Eder D. N. Wildschiodtz G. (1999). Nocturnal sweating and temperature in depression. Acta Psychiatr. Scand. 100, 295–301. 10.1111/j.1600-0447.1999.tb10864.x 10510699

[B15] Avery D. H. Wildschiodtz G. Rafaelsen O. J. (1982b). Nocturnal temperature in affective disorder. J. Affect. Disord. 4, 61–71. 10.1016/0165-0327(82)90020-9 6461688

[B16] Avery D. Wildschiodtz G. Rafaelsen O. (1982a). REM latency and temperature in affective disorder before and after treatment. Biol. Psychiatry 17, 463–470. 7082712

[B17] Bagg C. E. Crookes T. G. (1966). Palmar digital sweating in women suffering from depression. Br. J. Psychiatry 112, 1251–1255. 10.1192/bjp.112.493.1251 5966154

[B18] Bar K. J. Brehm S. Boettger M. K. Boettger S. Wagner G. Sauer H. (2005). Pain perception in major depression depends on pain modality. Pain 117, 97–103. 10.1016/j.pain.2005.05.016 16061323

[B19] Bar K. J. Terhaar J. Boettger M. K. Boettger S. Berger S. Weiss T. (2011). Pseudohypoalgesia on the skin: a novel view on the paradox of pain perception in depression. J. Clin. Psychopharmacol. 31, 103–107. 10.1097/JCP.0b013e3182046797 21192152

[B20] Barton D. A. Dawood T. Lambert E. A. Esler M. D. Haikerwal D. Brenchley C.. (2007). Sympathetic activity in major depressive disorder: identifying those at increased cardiac risk? J. Hypertens. 25, 2117–2124. 10.1097/HJH.0b013e32829baae7 17885556

[B21] Beever R. (2010). The effects of repeated thermal therapy on quality of life in patients with type II diabetes mellitus. J. Altern. Complement. Med. 16, 677–681. 10.1089/acm.2009.0358 20569036

[B22] Berglund L. H. Prytz H. S. Perski A. Svartberg J. (2011). Testosterone levels and psychological health status in men from a general population: the Tromso study. Aging Male 14, 37–41. 10.3109/13685538.2010.522276 20923289

[B23] Bested A. C. Logan A. C. Selhub E. M. (2013). Intestinal microbiota, probiotics and mental health: from Metchnikoff to modern advances: part III – convergence toward clinical trials. Gut Pathog. 5, 4. 10.1186/1757-4749-5-4 23497650 PMC3605358

[B24] Bifulco A. Moran P. M. Ball C. Bernazzani O. (2002a). Adult attachment style. I: its relationship to clinical depression. Soc. Psychiatry Psychiatr. Epidemiol. 37, 50–59. 10.1007/s127-002-8215-0 11931088

[B25] Bifulco A. Moran P. M. Ball C. Lillie A. (2002b). Adult attachment style. II: its relationship to psychosocial depressive-vulnerability. Soc. Psychiatry Psychiatr. Epidemiol. 37, 60–67. 10.1007/s127-002-8216-x 11931089

[B26] Bilderbeck A. C. McCabe C. Wakeley J. McGlone F. Harris T. Cowen P. J.. (2011). Serotonergic activity influences the cognitive appraisal of close intimate relationships in healthy adults. Biol. Psychiatry 69, 720–725. 10.1016/j.biopsych.2010.12.038 21396628

[B27] Bilderbeck A. C. Wakeley J. Godlewska B. R. McGlone F. Harris T. Cowen P. J.. (2014). Preliminary evidence that sub-chronic citalopram triggers the re-evaluation of value in intimate partnerships. Soc. Cogn. Affect. Neurosci. 9, 1419–1425. 10.1093/scan/nst135 23996287 PMC4158381

[B28] Blomqvist A. Zhang E. T. Craig A. D. (2000). Cytoarchitectonic and immunohistochemical characterization of a specific pain and temperature relay, the posterior portion of the ventral medial nucleus, in the human thalamus. Brain 123(Pt 3), 601–619. 10.1093/brain/123.3.601 10686182

[B29] Blumberg H. P. Martin A. Kaufman J. Leung H. C. Skudlarski P. Lacadie C.. (2003). Frontostriatal abnormalities in adolescents with bipolar disorder: preliminary observations from functional MRI. Am. J. Psychiatry 160, 1345–1347. 10.1176/appi.ajp.160.7.1345 12832254

[B30] Bowlby J. (1969). Attachment and Loss. London: Hogarth Press.

[B31] Brazelton T. B. (1990). Touch as a touchstone: summary of the round table, in Touch: The Foundation of Experience, Vol. 4, eds Barnard K. E. Brazelton T. B. (Madison, WI: International Universities Press), 561–566.

[B32] Breiter H. C. Aharon I. Kahneman D. Dale A. Shizgal P. (2001). Functional imaging of neural responses to expectancy and experience of monetary gains and losses. Neuron 30, 619–639. 10.1016/S0896-6273(01)00303-8 11395019

[B33] Brischoux F. Bonnet X. Shine R. (2009). Kleptothermy: an additional category of thermoregulation, and a possible example in sea kraits (*Laticauda laticaudata*, Serpentes). Biol. Lett. 5, 729–731. 10.1098/rsbl.2009.0550 19656862 PMC2828009

[B34] Bromundt V. Wirz-Justice A. Kyburz S. Opwis K. Dammann G. Cajochen C. (2013). Circadian sleep-wake cycles, well-being, and light therapy in borderline personality disorder. J. Pers. Disord. 27, 680–696. 10.1521/pedi_2012_26_057 22928852

[B35] Buhle J. T. Kober H. Ochsner K. N. Mende-Siedlecki P. Weber J. Hughes B. L.. (2013). Common representation of pain and negative emotion in the midbrain periaqueductal gray. Soc. Cogn. Affect. Neurosci. 8, 609–616. 10.1093/scan/nss038 22446299 PMC3739905

[B36] Bushnell M. C. Ceko M. Low L. A. (2013). Cognitive and emotional control of pain and its disruption in chronic pain. Nat. Rev. Neurosci. 14, 502–511. 10.1038/nrn3516 23719569 PMC4465351

[B37] Cannon W. B. (1915). Bodily Changes in Pain, Hunger, Fear and Rage. New York, NY: Appleton.

[B38] Carmichael S. T. Price J. L. (1995). Sensory and premotor connections of the orbital and medial prefrontal cortex of macaque monkeys. J. Comp. Neurol. 363, 642–664. 10.1002/cne.903630409 8847422

[B39] Carney R. M. Hong B. A. Kulkarni S. Kapila A. (1981). A comparison of EMG and SCL in normal and depressed subjects. Pavlov. J. Biol. Sci. 16, 212–216. 7329745 10.1007/BF03003361

[B40] Casey K. L. Minoshima S. Morrow T. J. Koeppe R. A. (1996). Comparison of human cerebral activation pattern during cutaneous warmth, heat pain, and deep cold pain. J. Neurophysiol. 76, 571–581. 8836245 10.1152/jn.1996.76.1.571

[B41] Cattaneo L. Chierici E. Cucurachi L. Cobelli R. Pavesi G. (2007). Posterior insular stroke causing selective loss of contralateral nonpainful thermal sensation. Neurology 68, 237. 10.1212/01.wnl.0000251310.71452.83 17224581

[B42] Cavada C. Company T. Tejedor J. Cruz-Rizzolo R. J. Reinoso-Suarez F. (2000). The anatomical connections of the macaque monkey orbitofrontal cortex. A review. Cereb. Cortex 10, 220–242. 10.1093/cercor/10.3.220 10731218

[B43] Christensen B. N. Perl E. R. (1970). Spinal neurons specifically excited by noxious or thermal stimuli: marginal zone of the dorsal horn. J. Neurophysiol. 33, 293–307. 5415075 10.1152/jn.1970.33.2.293

[B44] Craig A. D. (2003). Pain mechanisms: labeled lines versus convergence in central processing. Annu. Rev. Neurosci. 26, 1–30. 10.1146/annurev.neuro.26.041002.131022 12651967

[B45] Craig A. D. (2009). How do you feel–now? The anterior insula and human awareness. Nat. Rev. Neurosci. 10, 59–70. 10.1038/nrn2555 19096369

[B46] Craig A. D. Bushnell M. C. Zhang E. T. Blomqvist A. (1994). A thalamic nucleus specific for pain and temperature sensation. Nature 372, 770–773. 10.1038/372770a0 7695716

[B47] Craig A. D. Chen K. Bandy D. Reiman E. M. (2000). Thermosensory activation of insular cortex. Nat. Neurosci. 3, 184–190. 10.1038/72131 10649575

[B48] Craig A. D. Krout K. Andrew D. (2001). Quantitative response characteristics of thermoreceptive and nociceptive lamina I spinothalamic neurons in the cat. J. Neurophysiol. 86, 1459–1480. 11535691 10.1152/jn.2001.86.3.1459

[B49] Craig A. D. Zhang E. T. Blomqvist A. (1999). A distinct thermoreceptive subregion of lamina I in nucleus caudalis of the owl monkey. J. Comp. Neurol. 404, 221–234. 9934996

[B50] Critchley H. D. Nagai Y. Gray M. A. Mathias C. J. (2011). Dissecting axes of autonomic control in humans: insights from neuroimaging. Auton. Neurosci. 161, 34–42. 10.1016/j.autneu.2010.09.005 20926356

[B51] Cryan J. F. Dinan T. G. (2012). Mind-altering microorganisms: the impact of the gut microbiota on brain and behaviour. Nat. Rev. Neurosci. 13, 701–712. 10.1038/nrn3346 22968153

[B52] Davis K. D. Kwan C. L. Crawley A. P. Mikulis D. J. (1998). Functional MRI study of thalamic and cortical activations evoked by cutaneous heat, cold, and tactile stimuli. J. Neurophysiol. 80, 1533–1546. 9744957 10.1152/jn.1998.80.3.1533

[B53] Dawson M. E. Schell A. M. Braaten J. R. Catania J. J. (1985). Diagnostic utility of autonomic measures for major depressive disorders. Psychiatry Res. 15, 261–270. 10.1016/0165-1781(85)90063-0 3865244

[B54] Dawson M. E. Schell A. M. Catania J. J. (1977). Autonomic correlates of depression and clinical improvement following electroconvulsive shock therapy. Psychophysiology 14, 569–578. 10.1111/j.1469-8986.1977.tb01201.x 928608

[B55] Dawson M. E. Schell A. M. Filion D. L. (2007). The electrodermal system, in Handbook of Psychophysiology, eds Cacioppo J. J. Tassinary L. G. Berntson G. G. (Cambridge: Cambridge University Press), 159–181 10.1017/CBO9780511546396.007

[B56] Denenberg V. H. (1968). The effects of early experience, in The Behavior of Domestic Animals, ed Hafez E. S. E. (London: Bailliere, Tindall, and Cassell), 95–130.

[B57] Dietzel M. Saletu B. Lesch O. M. Sieghart W. Schjerve M. (1986). Light treatment in depressive illness. Polysomnographic, psychometric and neuroendocrinological findings. Eur. Neurol. 25(Suppl 2), 93–103. 10.1159/000116089 3758132

[B58] Donat D. C. McCullough J. P. (1983). Psychophysiological discriminants of depression at rest and in response to stress. J. Clin. Psychol. 39, 315–320. 6874964 10.1002/1097-4679(198305)39:3<315::aid-jclp2270390303>3.0.co;2-h

[B59] Donnellan M. B. Lucas R. E. Cesario J. (2014). On the association between loneliness and bathing habits: Nine replications of Bargh and Shalev 2012 study 1. Emotion. [Epub ahead of print]. 10.1037/a0036079 24821396

[B60] Dostrovsky J. O. Craig A. D. (1996). Cooling-specific spinothalamic neurons in the monkey. J. Neurophysiol. 76, 3656–3665. 8985864 10.1152/jn.1996.76.6.3656

[B61] Dostrovsky J. O. Hellon R. F. (1978). The representation of facial temperature in the caudal trigeminal nucleus of the cat. J. Physiol. 277, 29–47. 10.1113/jphysiol.1978.sp012258 650531 PMC1282375

[B62] Drevets W. C. Price J. L. Furey M. L. (2008). Brain structural and functional abnormalities in mood disorders: implications for neurocircuitry models of depression. Brain Struct. Funct. 213, 93–118. 10.1007/s00429-008-0189-x 18704495 PMC2522333

[B63] Duncan W. C. Jr. (1996). Circadian rhythms and the pharmacology of affective illness. Pharmacol. Ther. 71, 253–312. 10.1016/S0163-7258(96)00092-7 8940745

[B64] Edman G. Asberg M. Levander S. Schalling D. (1986). Skin conductance habituation and cerebrospinal fluid 5-hydroxyindoleacetic acid in suicidal patients. Arch. Gen. Psychiatry 43, 586–592. 10.1001/archpsyc.1986.01800060080010 2423049

[B65] Ellingsen D. M. Wessberg J. Chelnokova O. Olausson H. Laeng B. Leknes S. (2014). In touch with your emotions: oxytocin and touch change social impressions while others' facial expressions can alter touch. Psychoneuroendocrinology 39, 11–20. 10.1016/j.psyneuen.2013.09.017 24275000

[B66] El-Sheikh M. Arsiwalla D. D. (2011). Children's sleep, skin conductance level and mental health. J. Sleep Res. 20, 326–337. 10.1111/j.1365-2869.2010.00880.x 20727066

[B67] Engel G. L. Schmale A. H. (1972). Conservation withdrawal: a primary regulatory process for organic homeostasis, in Physiology, Emotions and Psychosomatic Illness, CIBA Foundation Symposium, Vol. 8, eds Porter R. Knight J. (New York, NY: Elsevier), 57–95. 10.1002/9780470719916.ch5 4144967

[B68] Eysenck H. J. (1967). The Biological Basis of Personality. Springfield, IL: Thomas.

[B69] Fahim I. Ismail M. Osman O. H. (1973). Role of 5-hydroxytryptamine in ketamine-induced hypothermia in the rat. Br. J. Pharmacol. 48, 570–576. 10.1111/j.1476-5381.1973.tb08243.x 4274691 PMC1776151

[B70] Farrell M. J. Trevaks D. McAllen R. M. (2014). Preoptic activation and connectivity during thermal sweating in humans. Temperature 1, 135–141 10.4161/temp.29667 PMC4977170 27583295

[B71] Fraguas R. Jr. Marci C. Fava M. Iosifescu D. V. Bankier B. Loh R.. (2007). Autonomic reactivity to induced emotion as potential predictor of response to antidepressant treatment. Psychiatry Res. 151, 169–172. 10.1016/j.psychres.2006.08.008 17360044

[B72] Franken I. H. Rassin E. Muris P. (2007). The assessment of anhedonia in clinical and non-clinical populations: further validation of the Snaith-Hamilton Pleasure Scale (SHAPS). J. Affect. Disord. 99, 83–89. 10.1016/j.jad.2006.08.020 16996138

[B73] Garcia-Larrea L. Perchet C. Creac'h C. Convers P. Peyron R. Laurent B.. (2010). Operculo-insular pain (parasylvian pain): a distinct central pain syndrome. Brain 133, 2528–2539. 10.1093/brain/awq220 20724291

[B74] Gardner K. L. Thrivikraman K. V. Lightman S. L. Plotsky P. M. Lowry C. A. (2005). Early life experience alters behavior during social defeat: focus on serotonergic systems. Neuroscience 136, 181–191. 10.1016/j.neuroscience.2005.07.042 16182451

[B75] Gauriau C. Bernard J. F. (2004). A comparative reappraisal of projections from the superficial laminae of the dorsal horn in the rat: the forebrain. J. Comp. Neurol. 468, 24–56. 10.1002/cne.10873 14648689

[B76] Gazzola V. Spezio M. L. Etzel J. A. Castelli F. Adolphs R. Keysers C. (2012). Primary somatosensory cortex discriminates affective significance in social touch. Proc. Natl. Acad. Sci. U.S.A. 109, E1657–E1666. 10.1073/pnas.1113211109 22665808 PMC3382530

[B77] Giltay E. J. Enter D. Zitman F. G. Penninx B. W. Van P. J. Spinhoven P.. (2012). Salivary testosterone: associations with depression, anxiety disorders, and antidepressant use in a large cohort study. J. Psychosom. Res. 72, 205–213. 10.1016/j.jpsychores.2011.11.014 22325700

[B78] Goetze U. Tolle R. (1987). Circadian rhythm of free urinary cortisol, temperature and heart rate in endogenous depressives and under antidepressant therapy. Neuropsychobiology 18, 175–184. 10.1159/000118414 3454423

[B79] Gottfried J. A. Deichmann R. Winston J. S. Dolan R. J. (2002). Functional heterogeneity in human olfactory cortex: an event-related functional magnetic resonance imaging study. J. Neurosci. 22, 10819–10828. 12486175 10.1523/JNEUROSCI.22-24-10819.2002 PMC6758422

[B80] Grabenhorst F. Rolls E. T. (2008). Selective attention to affective value alters how the brain processes taste stimuli. Eur. J. Neurosci. 27, 723–729. 10.1111/j.1460-9568.2008.06033.x 18279324

[B81] Grabenhorst F. Rolls E. T. Margot C. da Silva M. A. Velazco M. I. (2007). How pleasant and unpleasant stimuli combine in different brain regions: odor mixtures. J. Neurosci. 27, 13532–13540. 10.1523/JNEUROSCI.3337-07.2007 18057211 PMC6673088

[B82] Guest S. Grabenhorst F. Essick G. Chen Y. Young M. McGlone F.. (2007). Human cortical representation of oral temperature. Physiol. Behav. 92, 975–984. 10.1016/j.physbeh.2007.07.004 17689575

[B83] Hahn A. C. Whitehead R. D. Albrecht M. Lefevre C. E. Perrett D. I. (2012). Hot or not? Thermal reactions to social contact. Biol. Lett. 8, 864–867. 10.1098/rsbl.2012.0338 22647931 PMC3440979

[B84] Hale M. W. Dady K. F. Evans A. K. Lowry C. A. (2011). Evidence for *in vivo* thermosensitivity of serotonergic neurons in the rat dorsal raphe nucleus and raphe pallidus nucleus implicated in thermoregulatory cooling. Exp. Neurol. 227, 264–278. 10.1016/j.expneurol.2010.11.012 21111735

[B85] Hale M. W. Raison C. L. Lowry C. A. (2013). Integrative physiology of depression and antidepressant drug action: implications for serotonergic mechanisms of action and novel therapeutic strategies for treatment of depression. Pharmacol. Ther. 137, 108–118. 10.1016/j.pharmthera.2012.09.005 23017938

[B86] Hale M. W. Shekhar A. Lowry C. A. (2012). Stress-related serotonergic systems: implications for symptomatology of anxiety and affective disorders. Cell. Mol. Neurobiol. 32, 695–708. 10.1007/s10571-012-9827-1 22484834 PMC3378822

[B87] Hamilton J. P. Etkin A. Furman D. J. Lemus M. G. Johnson R. F. Gotlib I. H. (2012). Functional neuroimaging of major depressive disorder: a meta-analysis and new integration of base line activation and neural response data. Am. J. Psychiatry 169, 693–703. 10.1176/appi.ajp.2012.11071105 22535198 PMC11889638

[B88] Han Z. S. Zhang E. T. Craig A. D. (1998). Nociceptive and thermoreceptive lamina I neurons are anatomically distinct. Nat. Neurosci. 1, 218–225. 10.1038/665 10195146

[B89] Hanusch K. U. Janssen C. H. Billheimer D. Jenkins I. Spurgeon E. Lowry C. A.. (2013). Whole-body hyperthermia for the treatment of major depression: associations with thermoregulatory cooling. Am. J. Psychiatry 170, 802–804. 10.1176/appi.ajp.2013.12111395 23820835

[B90] Harlow H. (1958). The nature of love. Am. Psychol. 13, 673–685 10.1037/h0047884 4984312

[B91] Hicks C. Ramos L. Reekie T. Misagh G. H. Narlawar R. Kassiou M.. (2014). Body temperature and cardiac changes induced by peripherally administered oxytocin, vasopressin and the non-peptide oxytocin receptor agonist WAY 267,464: a biotelemetry study in rats. Br. J. Pharmacol. 171, 2868–2887. 10.1111/bph.12613 24641248 PMC4243861

[B92] Hilderink P. H. Burger H. Deeg D. J. Beekman A. T. Oude Voshaar R. C. (2012). The temporal relation between pain and depression: results from the longitudinal aging study Amsterdam. Psychosom. Med. 74, 945–951. 10.1097/PSY.0b013e3182733fdd 23115345

[B93] Hintikka J. Niskanen L. Koivumaa-Honkanen H. Tolmunen T. Honkalampi K. Lehto S. M.. (2009). Hypogonadism, decreased sexual desire, and long-term depression in middle-aged men. J. Sex. Med. 6, 2049–2057. 10.1111/j.1743-6109.2009.01299.x 19453895

[B94] Hong J. Sun Y. (2012). Warm it up with love: the effect of physical coldness on liking of romance movies. J. Consum. Res. 39, 293–306 10.1086/662613

[B95] Hua I. H. Strigo I. A. Baxter L. C. Johnson S. C. Craig A. D. (2005). Anteroposterior somatotopy of innocuous cooling activation focus in human dorsal posterior insular cortex. Am. J. Physiol. Regul. Integr. Comp. Physiol. 289, R319–R325. 10.1152/ajpregu.00123.2005 15805097

[B96] Hulvershorn L. A. Karne H. Gunn A. D. Hartwick S. L. Wang Y. Hummer T. A.. (2012). Neural activation during facial emotion processing in unmedicated bipolar depression, euthymia, and mania. Biol. Psychiatry 71, 603–610. 10.1016/j.biopsych.2011.10.038 22206876 PMC3703667

[B97] Iacono W. G. Lykken D. T. Haroian K. P. Peloquin L. J. Valentine R. H. Tuason V. B. (1984). Electrodermal activity in euthymic patients with affective disorders: one-year retest stability and the effects of stimulus intensity and significance. J. Abnorm. Psychol. 93, 304–311. 10.1037/0021-843X.93.3.304 6470315

[B98] Iacono W. G. Lykken D. T. Peloquin L. J. Lumry A. E. Valentine R. H. Tuason V. B. (1983). Electrodermal activity in euthymic unipolar and bipolar affective disorders. A possible marker for depression. Arch. Gen. Psychiatry 40, 557–565. 10.1001/archpsyc.1983.01790050083010 6838333

[B99] Ijzerman H. Gallucci M. Pouw W. T. Weibetagerber S. C. Van Doesum N. J. Williams K. D. (2012). Cold-blooded loneliness: social exclusion leads to lower skin temperatures. Acta Psychol. (Amst). 140, 283–288. 10.1016/j.actpsy.2012.05.002 22717422

[B100] Ijzerman H. Janssen J. Coan J. (2014). Maintaining Warm, Trusting Relationships with Brands: Increased Temperature Perceptions After Thinking of Communal Brands. SSRN. Available online at: http://ssrncom/abstract=2386029 (Accessed January 27, 2014). 10.1371/journal.pone.0125194 PMC4411151 25915686

[B101] Ijzerman H. Karremans J. C. Thomsen L. Schubert T. W. (2013). Caring for sharing: how attachment styles modulate communal cues of physical warmth. Soc. Psychol. 44, 160–166 10.1027/1864-9335/a000142

[B102] Ijzerman H. Semin G. R. (2009). The thermometer of social relations: mapping social proximity on temperature. Psychol. Sci. 20, 1214–1220. 10.1111/j.1467-9280.2009.02434.x 19732385

[B103] Ijzerman H. Semin G. R. (2010). Temperature perceptions as a ground for social proximity. J. Exp. Soc. Psychol. 46, 867–873. 10.1016/j.jesp.2010.07.01519732385

[B104] Inagaki T. K. Eisenberger N. I. (2013). Shared neural mechanisms underlying social warmth and physical warmth. Psychol. Sci. 24, 2272–2280. 10.1177/0956797613492773 24048423

[B105] Insel T. R. Sahakian B. J. (2012). Drug research: a plan for mental illness. Nature 483, 269. 10.1038/483269a 22422245

[B106] Ioannou S. Ebisch S. Aureli T. Bafunno D. Ioannides H. A. Cardone D.. (2013). The autonomic signature of guilt in children: a thermal infrared imaging study. PLoS ONE 8:e79440. 10.1371/journal.pone.0079440 24260220 PMC3834185

[B107] Ioannou S. Morris P. Mercer H. Baker M. Gallese V. Reddy V. (2014). Proximity and gaze influences facial temperature: a thermal infrared imaging study. Front. Psychol. 5:845. 10.3389/fpsyg.2014.00845 25136326 PMC4120854

[B108] Kadohisa M. Rolls E. T. Verhagen J. V. (2004). Orbitofrontal cortex: neuronal representation of oral temperature and capsaicin in addition to taste and texture. Neuroscience 127, 207–221. 10.1016/j.neuroscience.2004.04.037 15219683

[B109] Kang Y. Williams L. E. Clark M. S. Gray J. R. Bargh J. A. (2011). Physical temperature effects on trust behavior: the role of insula. Soc. Cogn. Affect. Neurosci. 6, 507–515. 10.1093/scan/nsq077 20802090 PMC3150863

[B110] Karama S. Lecours A. R. Leroux J. M. Bourgouin P. Beaudoin G. Joubert S.. (2002). Areas of brain activation in males and females during viewing of erotic film excerpts. Hum. Brain Mapp. 16, 1–13. 10.1002/hbm.10014 11870922 PMC6871831

[B111] Keay K. A. Bandler R. (2001). Parallel circuits mediating distinct emotional coping reactions to different types of stress. Neurosci. Biobehav. Rev. 25, 669–678. 10.1016/S0149-7634(01)00049-5 11801292

[B112] Kelly K. J. Donner N. C. Hale M. W. Lowry C. A. (2011). Swim stress activates serotonergic and nonserotonergic neurons in specific subdivisions of the rat dorsal raphe nucleus in a temperature-dependent manner. Neuroscience 197, 251–268. 10.1016/j.neuroscience.2011.09.011 21945646 PMC3219528

[B113] Kemp A. H. Stephan B. C. Hopkinson P. Sumich A. L. Paul R. H. Clark C. R.. (2005). Toward an integrated profile of depression: evidence from the brain resource international database. J. Integr. Neurosci. 4, 95–106. 10.1142/S0219635205000665 16035143

[B114] Kiser D. Steemers B. Branchi I. Homberg J. R. (2012). The reciprocal interaction between serotonin and social behaviour. Neurosci. Biobehav. Rev. 36, 786–798. 10.1016/j.neubiorev.2011.12.009 22206901

[B115] Kistler A. Mariauzouls C. Von B. K. (1998). Fingertip temperature as an indicator for sympathetic responses. Int. J. Psychophysiol. 29, 35–41. 10.1016/S0167-8760(97)00087-1 9641246

[B116] Koltyn K. F. Robins H. I. Schmitt C. L. Cohen J. D. Morgan W. P. (1992). Changes in mood state following whole-body hyperthermia. Int. J. Hyperthermia 8, 305–307. 10.3109/02656739209021785 1607735

[B117] Koolhaas J. M. Korte S. M. De Boer S. F. Van D. V. Van Reenen C. G. Hopster H.. (1999). Coping styles in animals: current status in behavior and stress-physiology. Neurosci. Biobehav. Rev. 23, 925–935. 10.1016/S0149-7634(99)00026-3 10580307

[B118] Korner A. F. Thoman E. B. (1972). The relative efficacy of contact and vestibular-proprioceptive stimulation in soothing neonates. Child Dev. 43, 443–453. 10.2307/1127547 4537551

[B119] Kranz F. Ishai A. (2006). Face perception is modulated by sexual preference. Curr. Biol. 16, 63–68. 10.1016/j.cub.2005.10.070 16401423

[B120] Kringelbach M. L. Rolls E. T. (2004). The functional neuroanatomy of the human orbitofrontal cortex: evidence from neuroimaging and neuropsychology. Prog. Neurobiol. 72, 341–372. 10.1016/j.pneurobio.2004.03.006 15157726

[B121] Krout K. E. Belzer R. E. Loewy A. D. (2002). Brainstem projections to midline and intralaminar thalamic nuclei of the rat. J. Comp. Neurol. 448, 53–101. 10.1002/cne.10236 12012375

[B122] Kuraoka K. Nakamura K. (2011). The use of nasal skin temperature measurements in studying emotion in macaque monkeys. Physiol. Behav. 102, 347–355. 10.1016/j.physbeh.2010.11.029 21130103

[B123] Lally N. Nugent A. C. Luckenbaugh D. A. Ameli R. Roiser J. P. Zarate C. A. (2014). Anti-anhedonic effect of ketamine and its neural correlates in treatment-resistant bipolar depression. Transl. Psychiatry 4, e469. 10.1038/tp.2014.105 25313512 PMC4350513

[B124] LeBel E. P. Campbell L. (2013). Heightened sensitivity to temperature cues in individuals with high anxious attachment: real or elusive phenomenon? Psychol. Sci. 24, 2128–2130. 10.1177/0956797613486983 23990197

[B125] Lee H. S. Kim M. A. Valentino R. J. Waterhouse B. D. (2003). Glutamatergic afferent projections to the dorsal raphe nucleus of the rat. Brain Res. 963, 57–71. 10.1016/S0006-8993(02)03841-6 12560111

[B126] Levendosky A. A. Josep-Vanderpool J. R. Hardin T. Sorek E. Rosenthal N. E. (1991). Core body temperature in patients with seasonal affective disorder and normal controls in summer and winter. Biol. Psychiatry 29, 524–534. 10.1016/0006-3223(91)90089-5 2054429

[B127] Leventhal A. M. Chasson G. S. Tapia E. Miller E. K. Pettit J. W. (2006). Measuring hedonic capacity in depression: a psychometric analysis of three anhedonia scales. J. Clin. Psychol. 62, 1545–1558. 10.1002/jclp.20327 17019674

[B128] Lindgren L. Westling G. Brulin C. Lehtipalo S. Andersson M. Nyberg L. (2012). Pleasant human touch is represented in pregenual anterior cingulate cortex. Neuroimage 59, 3427–3432. 10.1016/j.neuroimage.2011.11.013 22100768

[B129] Linke J. King A. V. Rietschel M. Strohmaier J. Hennerici M. Gass A.. (2012). Increased medial orbitofrontal and amygdala activation: evidence for a systems-level endophenotype of bipolar I disorder. Am. J. Psychiatry 169, 316–325. 10.1176/appi.ajp.2011.11050711 22267184

[B130] Loken L. S. Wessberg J. Morrison I. McGlone F. Olausson H. (2009). Coding of pleasant touch by unmyelinated afferents in humans. Nat. Neurosci. 12, 547–548. 10.1038/nn.2312 19363489

[B131] Lovick T. A. (1993). The periaqueductal gray-rostral medulla connection in the defence reaction: efferent pathways and descending control mechanisms. Behav. Brain Res. 58, 19–25. 10.1016/0166-4328(93)90087-7 8136045

[B132] Lowry C. A. Evans A. K. Gasser P. J. Hale M. W. Staub D. R. Shekhar A. (2008a). Topographical organization and chemoarchitecture of the dorsal raphe nucleus and the median raphe nucleus, in Serotonin and Sleep: Molecular, Functional and Clinical Aspects, eds Monti J. M. Pandi-Perumal B. L. Jacobs B. L. Nutt D. L. (Basel: Birkhauser) 25–68. 10.1007/978-3-7643-8561-3_2

[B133] Lowry C. A. Hale M. W. (2010). Serotonin and the neurobiology of anxious states, in Handbook of the Behavioral Neurobiology of Serotonin, eds Mûller C. P. Jacobs B. L. (Amsterdam: Elsevier), 379–398 10.1016/S1569-7339(10)70091-6

[B134] Lowry C. A. Hale M. W. Evans A. K. Heerkens J. Staub D. R. Gasser P. J.. (2008b). Serotonergic systems, anxiety, and affective disorder: focus on the dorsomedial part of the dorsal raphe nucleus. Ann. N. Y. Acad. Sci. 1148, 86–94. 10.1196/annals.1410.004 19120094

[B135] Lowry C. A. Hollis J. H. De V. A. Pan B. Brunet L. R. Hunt J. R.. (2007). Identification of an immune-responsive mesolimbocortical serotonergic system: potential role in regulation of emotional behavior. Neuroscience 146, 756–772. 10.1016/j.neuroscience.2007.01.067 17367941 PMC1868963

[B136] Lowry C. A. Johnson P. L. Hay-Schmidt A. Mikkelsen J. Shekhar A. (2005). Modulation of anxiety circuits by serotonergic systems. Stress 8, 233–246. 10.1080/10253890500492787 16423712

[B137] Maihofner C. Kaltenhauser M. Neundorfer B. Lang E. (2002). Temporo-spatial analysis of cortical activation by phasic innocuous and noxious cold stimuli–a magnetoencephalographic study. Pain 100, 281–290. 10.1016/S0304-3959(02)00276-2 12467999

[B138] Man M. S. Mikheenko Y. Braesicke K. Cockcroft G. Roberts A. C. (2012). Serotonin at the level of the amygdala and orbitofrontal cortex modulates distinct aspects of positive emotion in primates. Int. J. Neuropsychopharmacol 15, 91–105. 10.1017/S1461145711000587 21726490 PMC3243904

[B139] Marchand W. R. Yurgelun-Todd D. (2010). Striatal structure and function in mood disorders: a comprehensive review. Bipolar Disord. 12, 764–785. 10.1111/j.1399-5618.2010.00874.x 21176024

[B140] Marcy T. R. Britton M. L. (2005). Antidepressant-induced sweating. Ann. Pharmacother. 39, 748–752. 10.1345/aph.1E564 15728327

[B141] McCabe C. Rolls E. T. (2007). Umami: a delicious flavor formed by convergence of taste and olfactory pathways in the human brain. Eur. J. Neurosci. 25, 1855–1864. 10.1111/j.1460-9568.2007.05445.x 17432971

[B142] McCabe C. Rolls E. T. Bilderbeck A. McGlone F. (2008). Cognitive influences on the affective representation of touch and the sight of touch in the human brain. Soc. Cogn. Affect. Neurosci. 3, 97–108. 10.1093/scan/nsn005 19015100 PMC2555465

[B144] McCabe C. Woffindale C. Harmer C. J. Cowen P. J. (2012). Neural processing of reward and punishment in young people at increased familial risk of depression. Biol. Psychiatry 72, 588–594. 10.1016/j.biopsych.2012.04.034 22704059

[B145] McCarron L. T. (1973). Psychophysiological discriminants of reactive depression. Psychophysiology 10, 223–230. 10.1111/j.1469-8986.1973.tb00520.x 4145000

[B146] McCleskey E. W. (1997). Thermoreceptors: recent heat in thermosensation. Curr. Biol. 7, R679–R681. 10.1016/S0960-9822(06)00354-X 9382796

[B147] McGlone F. Wessberg J. Olausson H. (2014). Discriminative and affective touch: sensing and feeling. Neuron 82, 737–755. 10.1016/j.neuron.2014.05.001 24853935

[B148] Merla A. Romani G. L. (2007). Thermal signatures of emotional arousal: a functional infrared imaging study. Conf. Proc. IEEE Eng. Med. Biol. Soc. 2007, 247–249. 10.1109/IEMBS.2007.4352270 18001936

[B149] Mirkin A. M. Coppen A. (1980). Electrodermal activity in depression: clinical and biochemical correlates. Br. J. Psychiatry 137, 93–97. 10.1192/bjp.137.1.93 7459546

[B150] Morecraft R. J. Geula C. Mesulam M. M. (1992). Cytoarchitecture and neural afferents of orbitofrontal cortex in the brain of the monkey. J. Comp. Neurol. 323, 341–358. 10.1002/cne.903230304 1460107

[B151] Moussaieff A. Rimmerman N. Bregman T. Straiker A. Felder C. C. Shoham S.. (2008). Incensole acetate, an incense component, elicits psychoactivity by activating TRPV3 channels in the brain. FASEB J. 22, 3024–3034. 10.1096/fj.07-101865 18492727 PMC2493463

[B152] Nagai Y. Critchley H. D. Featherstone E. Trimble M. R. Dolan R. J. (2004). Activity in ventromedial prefrontal cortex covaries with sympathetic skin conductance level: a physiological account of a “default mode” of brain function. Neuroimage 22, 243–251. 10.1016/j.neuroimage.2004.01.019 15110014

[B153] Nakamura K. Morrison S. F. (2008). A thermosensory pathway that controls body temperature. Nat. Neurosci. 11, 62–71. 10.1038/nn2027 18084288 PMC2423341

[B154] Nakamura K. Morrison S. F. (2010). A thermosensory pathway mediating heat-defense responses. Proc. Natl. Acad. Sci. U.S.A. 107, 8848–8853. 10.1073/pnas.0913358107 20421477 PMC2889337

[B155] Nakayama K. Goto S. Kuraoka K. Nakamura K. (2005). Decrease in nasal temperature of rhesus monkeys (*Macaca mulatta*) in negative emotional state. Physiol. Behav. 84, 783–790. 10.1016/j.physbeh.2005.03.009 15885256

[B156] Nakonezny P. A. Carmody T. J. Morris D. W. Kurian B. T. Trivedi M. H. (2010). Psychometric evaluation of the Snaith-Hamilton pleasure scale in adult outpatients with major depressive disorder. Int. Clin. Psychopharmacol. 25, 328–333. 10.1097/YIC.0b013e32833eb5ee 20805756 PMC2957191

[B157] Nimbalkar S. M. Patel V. K. Patel D. V. Nimbalkar A. S. Sethi A. Phatak A. (2014). Effect of early skin-to-skin contact following normal delivery on incidence of hypothermia in neonates more than 1800 g: randomized control trial. J. Perinatol. 34, 364–368. 10.1038/jp.2014.15 24556982

[B158] Nordin M. (1990). Low-threshold mechanoreceptive and nociceptive units with unmyelinated (C) fibres in the human supraorbital nerve. J. Physiol. 426, 229–240. 10.1113/jphysiol.1990.sp018135 2231398 PMC1189885

[B159] O'Doherty J. Kringelbach M. L. Rolls E. T. Hornak J. Andrews C. (2001). Abstract reward and punishment representations in the human orbitofrontal cortex. Nat. Neurosci. 4, 95–102. 10.1038/82959 11135651

[B160] O'Doherty J. Winston J. Critchley H. Perrett D. Burt D. M. Dolan R. J. (2003). Beauty in a smile: the role of medial orbitofrontal cortex in facial attractiveness. Neuropsychologia 41, 147–155. 10.1016/S0028-3932(02)00145-8 12459213

[B161] Orme J. G. Reis J. Herz E. J. (1986). Factorial and discriminant validity of the Center for Epidemiological Studies Depression (CES-D) scale. J. Clin. Psychol. 42, 28–33. 3950011 10.1002/1097-4679(198601)42:1<28::aid-jclp2270420104>3.0.co;2-t

[B162] Papolos D. F. Teicher M. H. Faedda G. L. Murphy P. Mattis S. (2013). Clinical experience using intranasal ketamine in the treatment of pediatric bipolar disorder/fear of harm phenotype. J. Affect. Disord. 147, 431–436. 10.1016/j.jad.2012.08.040 23200737

[B163] Papolos D. Mattis S. Golshan S. Molay F. (2009). Fear of harm, a possible phenotype of pediatric bipolar disorder: a dimensional approach to diagnosis for genotyping psychiatric syndromes. J. Affect. Disord. 118, 28–38. 10.1016/j.jad.2009.06.016 19631388

[B164] Pariante C. M. Lightman S. L. (2008). The HPA axis in major depression: classical theories and new developments. Trends Neurosci. 31, 464–468. 10.1016/j.tins.2008.06.006 18675469

[B165] Parker G. Crawford J. (2007). Chocolate craving when depressed: a personality marker. Br. J. Psychiatry 191, 351–352. 10.1192/bjp.bp.106.033746 17906246

[B166] Parry B. L. LeVeau B. Mostofi N. Naham H. C. Loving R. Clopton P.. (1997). Temperature circadian rhythms during the menstrual cycle and sleep deprivation in premenstrual dysphoric disorder and normal comparison subjects. J. Biol. Rhythms 12, 34–46. 10.1177/074873049701200106 9104689

[B167] Parry B. L. Mendelson W. B. Duncan W. C. Sack D. A. Wehr T. A. (1989). Longitudinal sleep EEG, temperature, and activity measurements across the menstrual cycle in patients with premenstrual depression and in age-matched controls. Psychiatry Res. 30, 285–303. 10.1016/0165-1781(89)90020-6 2616693

[B168] Patapoutian A. Peier A. M. Story G. M. Viswanath V. (2003). ThermoTRP channels and beyond: mechanisms of temperature sensation. Nat. Rev. Neurosci. 4, 529–539. 10.1038/nrn1141 12838328

[B169] Paul E. D. Hale M. W. Lukkes J. L. Valentine M. J. Sarchet D. M. Lowry C. A. (2011). Repeated social defeat increases reactive emotional coping behavior and alters functional responses in serotonergic neurons in the rat dorsal raphe nucleus. Physiol. Behav. 104, 272–282. 10.1016/j.physbeh.2011.01.006 21238469 PMC3089807

[B170] Pavlidis I. Tsiamyrtzis P. Shastri D. Wesley A. Zhou Y. Lindner P.. (2012). Fast by nature – how stress patterns define human experience and performance in dexterous tasks. Sci. Rep. 2, 305. 10.1038/srep00305 22396852 PMC3294268

[B171] Pietersen C. Y. Bosker F. J. Postema F. Fokkema D. S. Korf J. Den Boer J. A. (2006). Ketamine administration disturbs behavioural and distributed neural correlates of fear conditioning in the rat. Prog. Neuropsychopharmacol. Biol. Psychiatry 30, 1209–1218. 10.1016/j.pnpbp.2006.02.019 16626845

[B172] Plassmann H. O'Doherty J. Rangel A. (2007). Orbitofrontal cortex encodes willingness to pay in everyday economic transactions. J. Neurosci. 27, 9984–9988. 10.1523/JNEUROSCI.2131-07.2007 17855612 PMC6672655

[B173] Ponseti J. Bosinski H. A. Wolff S. Peller M. Jansen O. Mehdorn H. M.. (2006). A functional endophenotype for sexual orientation in humans. Neuroimage 33, 825–833. 10.1016/j.neuroimage.2006.08.002 16979350

[B174] Porrino L. J. Goldman-Rakic P. S. (1982). Brainstem innervation of prefrontal and anterior cingulate cortex in the rhesus monkey revealed by retrograde transport of HRP. J. Comp. Neurol. 205, 63–76. 10.1002/cne.902050107 6121826

[B175] Rainville P. Duncan G. H. Price D. D. Carrier B. Bushnell M. C. (1997). Pain affect encoded in human anterior cingulate but not somatosensory cortex. Science 277, 968–971. 10.1126/science.277.5328.968 9252330

[B177] Raison C. L. Rutherford R. E. Woolwine B. J. Shuo C. Schettler P. Drake D. F.. (2013). A randomized controlled trial of the tumor necrosis factor antagonist infliximab for treatment-resistant depression: the role of baseline inflammatory biomarkers. JAMA Psychiatry 70, 31–41. 10.1001/2013.jamapsychiatry.4 22945416 PMC4015348

[B178] Raleigh M. J. Brammer G. L. Yuwiler A. Flannery J. W. McGuire M. T. Geller E. (1980). Serotonergic influences on the social behavior of vervet monkeys (*Cercopithecus aethiops* sabaeus). Exp. Neurol. 68, 322–334. 10.1016/0014-4886(80)90089-8 6444893

[B179] Raleigh M. J. McGuire M. T. Brammer G. L. Pollack D. B. Yuwiler A. (1991). Serotonergic mechanisms promote dominance acquisition in adult male vervet monkeys. Brain Res. 559, 181–190. 10.1016/0006-8993(91)90001-C 1794096

[B180] Rausch J. L. Johnson M. E. Corley K. M. Hobby H. M. Shendarkar N. Fei Y.. (2003). Depressed patients have higher body temperature: 5-HT transporter long promoter region effects. Neuropsychobiology 47, 120–127. 10.1159/000070579 12759553

[B181] Raymann R. J. Swaab D. F. Van Someren E. J. (2005). Cutaneous warming promotes sleep onset. Am. J. Physiol. Regul. Integr. Comp. Physiol. 288, R1589–R1597. 10.1152/ajpregu.00492.2004 15677527

[B182] Raymann R. J. Swaab D. F. Van Someren E. J. (2008). Skin deep: enhanced sleep depth by cutaneous temperature manipulation. Brain 131, 500–513. 10.1093/brain/awm315 18192289

[B183] Re D. E. Whitehead R. D. Xiao D. Perrett D. I. (2011). Oxygenated-blood colour change thresholds for perceived facial redness, health, and attractiveness. PLoS ONE 6:e17859. 10.1371/journal.pone.0017859 21448270 PMC3063159

[B184] Redoute J. Stoleru S. Gregoire M. C. Costes N. Cinotti L. Lavenne F.. (2000). Brain processing of visual sexual stimuli in human males. Hum. Brain Mapp. 11, 162–177. 10.1002/1097-0193(200011)11:3<162::AID-HBM30>3.0.CO;2-A 11098795 PMC6871964

[B185] Rilling J. K. Young L. J. (2014). The biology of mammalian parenting and its effect on offspring social development. Science 345, 771–776. 10.1126/science.1252723 25124431 PMC4306567

[B186] Roberts A. C. (2011). The importance of serotonin for orbitofrontal function. Biol. Psychiatry 69, 1185–1191. 10.1016/j.biopsych.2010.12.037 21353665

[B187] Rolls E. T. (2005). Emotion Explained. Oxford: Oxford University Press 10.1093/acprof:oso/9780198570035.001.0001

[B188] Rolls E. T. (2009). The anterior and midcingulate cortices and reward, in Cingulate Neurobiology and Disease, ed Vogt B. A. (Oxford: Oxford University Press), 191–206.

[B189] Rolls E. T. (2010). The affective and cognitive processing of touch, oral texture, and temperature in the brain. Neurosci. Biobehav. Rev. 34, 237–245. 10.1016/j.neubiorev.2008.03.010 18468687

[B191] Rolls E. T. Grabenhorst F. Parris B. A. (2008). Warm pleasant feelings in the brain. Neuroimage 41, 1504–1513. 10.1016/j.neuroimage.2008.03.005 18468458

[B192] Rolls E. T. Kringelbach M. L. de Araujo I. E. (2003a). Different representations of pleasant and unpleasant odours in the human brain. Eur. J. Neurosci. 18, 695–703. 10.1046/j.1460-9568.2003.02779.x 12911766

[B193] Rolls E. T. McCabe C. (2007). Enhanced affective brain representations of chocolate in cravers vs. non-cravers. Eur. J. Neurosci. 26, 1067–1076. 10.1111/j.1460-9568.2007.05724.x 17714197

[B194] Rolls E. T. O'Doherty J. Kringelbach M. L. Francis S. Bowtell R. McGlone F. (2003b). Representations of pleasant and painful touch in the human orbitofrontal and cingulate cortices. Cereb. Cortex 13, 308–317. 10.1093/cercor/13.3.308 12571120

[B195] Romeijn N. Raymann R. J. Most E. Te L. B. Van Der Meijden W. P. Fronczek R.. (2011). Sleep, vigilance, and thermosensitivity. Pflugers Arch. 463, 169–176. 10.1007/s00424-011-1042-2 22048563 PMC3256315

[B196] Ross H. E. Young L. J. (2009). Oxytocin and the neural mechanisms regulating social cognition and affiliative behavior. Front. Neuroendocrinol. 30, 534–547. 10.1016/j.yfrne.2009.05.004 19481567 PMC2748133

[B197] Roy M. Shohamy D. Wager T. D. (2012). Ventromedial prefrontal-subcortical systems and the generation of affective meaning. Trends Cogn. Sci. 16, 147–156. 10.1016/j.tics.2012.01.005 22310704 PMC3318966

[B198] Rozeske R. R. Evans A. K. Frank M. G. Watkins L. R. Lowry C. A. Maier S. F. (2011). Uncontrollable, but not controllable, stress desensitizes 5-HT1A receptors in the dorsal raphe nucleus. J. Neurosci. 31, 14107–14115. 10.1523/JNEUROSCI.3095-11.2011 21976495 PMC3207271

[B199] Saper C. B. Loewy A. D. (1980). Efferent connections of the parabrachial nucleus in the rat. Brain Res. 197, 291–317. 10.1016/0006-8993(80)91117-8 7407557

[B200] Satpute A. B. Wager T. D. Cohen-Adad J. Bianciardi M. Choi J. K. Buhle J. T.. (2013). Identification of discrete functional subregions of the human periaqueductal gray. Proc. Natl. Acad. Sci. U.S.A. 110, 17101–17106. 10.1073/pnas.1306095110 24082116 PMC3801046

[B201] Scalco A. Z. Scalco M. Z. Azul J. B. Lotufo N. F. (2005). Hypertension and depression. Clinics (Sao. Paulo). 60, 241–250. 10.1590/S1807-59322005000300010 15962086

[B202] Schilder J. D. Ijzerman H. Denissen J. J. A. (2014). Physical Warmth and Perceptual Focus: A Replication of IJzerman and Semin (2009). SSRN. Available online at: http://ssrncom/abstract=2378184 (Accessed January 13, 2014). 10.1371/journal.pone.0112772 PMC4234632 25402343

[B203] Schwartz P. J. Murphy D. L. Wehr T. A. Garcia-Borreguero D. Oren D. A. Moul D. E.. (1997a). Effects of meta-chlorophenylpiperazine infusions in patients with seasonal affective disorder and healthy control subjects. Diurnal responses and nocturnal regulatory mechanisms. Arch. Gen. Psychiatry 54, 375–385. 10.1001/archpsyc.1997.01830160103013 9107154

[B204] Schwartz P. J. Rosenthal N. E. Turner E. H. Drake C. L. Liberty V. Wehr T. A. (1997b). Seasonal variation in core temperature regulation during sleep in patients with winter seasonal affective disorder. Biol. Psychiatry 42, 122–131. 10.1016/S0006-3223(96)00332-0 9209729

[B205] Schwier C. Kliem A. Boettger M. K. Bar K. J. (2010). Increased cold-pain thresholds in major depression. J. Pain 11, 287–290. 10.1016/j.jpain.2009.07.012 19944649

[B206] Severino S. K. Wagner D. R. Moline M. L. Hurt S. W. Pollak C. P. Zendell S. (1991). High nocturnal body temperature in premenstrual syndrome and late luteal phase dysphoric disorder. Am. J. Psychiatry 148, 1329–1335. 10.1176/ajp.148.10.1329 1897612

[B207] Shechter A. Lesperance P. Ng Ying Kin N. M. Boivin D. B. (2012). Nocturnal polysomnographic sleep across the menstrual cycle in premenstrual dysphoric disorder. Sleep Med. 13, 1071–1078. 10.1016/j.sleep.2012.05.012 22749440

[B208] Shinba T. (2014). Altered autonomic activity and reactivity in depression revealed by heart-rate variability measurement during rest and task conditions. Psychiatry Clin. Neurosci. 68, 225–233. 10.1111/pcn.12123 24313703

[B209] Shores M. M. Moceri V. M. Sloan K. L. Matsumoto A. M. Kivlahan D. R. (2005). Low testosterone levels predict incident depressive illness in older men: effects of age and medical morbidity. J. Clin. Psychiatry 66, 7–14. 10.4088/JCP.v66n0102 15669883

[B210] Sliz D. Hayley S. (2012). Major depressive disorder and alterations in insular cortical activity: a review of current functional magnetic imaging research. Front. Hum. Neurosci. 6:323. 10.3389/fnhum.2012.00323 23227005 PMC3512092

[B211] Small D. M. (2012). Flavor is in the brain. Physiol. Behav. 107, 540–552. 10.1016/j.physbeh.2012.04.011 22542991

[B212] Small D. M. Prescott J. (2005). Odor/taste integration and the perception of flavor. Exp. Brain Res. 166, 345–357. 10.1007/s00221-005-2376-9 16028032

[B213] Souetre E. Salvati E. Wehr T. A. Sack D. A. Krebs B. Darcourt G. (1988). Twenty-four-hour profiles of body temperature and plasma TSH in bipolar patients during depression and during remission and in normal control subjects. Am. J. Psychiatry 145, 1133–1137. 10.1176/ajp.145.9.1133 3414857

[B215] Stancak A. Mlynar J. Polacek H. Vrana J. (2006). Source imaging of the cortical 10 Hz oscillations during cooling and warming in humans. Neuroimage 33, 660–671. 10.1016/j.neuroimage.2006.06.049 16952469

[B216] Stephen I. D. Coetzee V. Law S. M. Perrett D. I. (2009). Skin blood perfusion and oxygenation colour affect perceived human health. PLoS ONE 4:e5083. 10.1371/journal.pone.0005083 19337378 PMC2659803

[B217] Storrie M. C. Doerr H. O. Johnson M. H. (1981). Skin conductance characteristics of depressed subjects before and after therapeutic intervention. J. Nerv. Ment. Dis. 169, 176–179. 10.1097/00005053-198103000-00004 7205243

[B218] Strigo I. A. Simmons A. N. Matthews S. C. Craig A. D. Paulus M. P. (2008). Increased affective bias revealed using experimental graded heat stimuli in young depressed adults: evidence of “emotional allodynia.” Psychosom. Med. 70, 338–344. 10.1097/PSY.0b013e3181656a48 18378870 PMC2742693

[B219] Sugiura Y. (1996). Spinal organization of C-fiber afferents related with nociception or non-nociception. Prog. Brain Res. 113, 320–339. 10.1016/S0079-6123(08)61096-1 9009743

[B220] Szuba M. P. Guze B. H. Baxter L. R. Jr. (1997). Electroconvulsive therapy increases circadian amplitude and lowers core body temperature in depressed subjects. Biol. Psychiatry 42, 1130–1137. 10.1016/S0006-3223(97)00046-2 9426883

[B221] Terrien J. Perret M. Aujard F. (2011). Behavioral thermoregulation in mammals: a review. Front. Biosci. 16:1428–1444. 10.2741/3797 21196240

[B222] Thorell L. H. (2009). Valid electrodermal hyporeactivity for depressive suicidal propensity offers links to cognitive theory. Acta Psychiatr. Scand. 119, 338–349. 10.1111/j.1600-0447.2009.01364.x 19245680

[B223] Thorell L. H. Kjellman B. F. D'elia G. (1987). Electrodermal activity in antidepressant medicated and unmedicated depressive patients and in matched healthy subjects. Acta Psychiatr. Scand. 76, 684–692. 10.1111/j.1600-0447.1987.tb02940.x 3442260

[B224] Tillet Y. (1992). Serotoninergic projections from the raphe nuclei to the preoptic area in sheep as revealed by immunohistochemistry and retrograde labeling. J. Comp. Neurol. 320, 267–272. 10.1002/cne.903200210 1619053

[B225] Tillet Y. Batailler M. Thibault J. (1993). Neuronal projections to the medial preoptic area of the sheep, with special reference to monoaminergic afferents-immunohistochemical and retrograde tract tracing studies. J. Comp. Neurol. 330, 195–220. 10.1002/cne.903300205 8491868

[B226] Tracey I. Iannetti G. D. (2006). Brainstem functional imaging in humans. Suppl. Clin. Neurophysiol. 58, 52–67. 10.1016/S1567-424X(09)70059-5 16623322

[B227] Tulving E. Schacter D. L. (1990). Priming and human memory systems. Science 247, 301–306. 10.1126/science.2296719 2296719

[B228] Ushinsky A. Reinhardt L. E. Simmons A. N. Strigo I. A. (2013). Further evidence of emotional allodynia in unmedicated young adults with major depressive disorder. PLoS ONE 8:e80507. 10.1371/journal.pone.0080507 24312229 PMC3842925

[B229] Uvnas-Moberg K. Bruzelius G. Alster P. Lundeberg T. (1993). The antinociceptive effect of non-noxious sensory stimulation is mediated partly through oxytocinergic mechanisms. Acta Physiol. Scand. 149, 199–204. 10.1111/j.1748-1716.1993.tb09612.x 8266809

[B230] Van Bockstaele E. J. Biswas A. Pickel V. M. (1993). Topography of serotonin neurons in the dorsal raphe nucleus that send axon collaterals to the rat prefrontal cortex and nucleus accumbens. Brain Res. 624, 188–198. 10.1016/0006-8993(93)90077-Z 8252391

[B231] Van Marken Lichtenbelt W. D. Daanen H. A. Wouters L. Fronczek R. Raymann R. J. Severens N. M.. (2006). Evaluation of wireless determination of skin temperature using iButtons. Physiol. Behav. 88, 489–497. 10.1016/j.physbeh.2006.04.026 16797616

[B232] Van Someren E. J. (2006). Mechanisms and functions of coupling between sleep and temperature rhythms. Prog. Brain Res. 153, 309–324. 10.1016/S0079-6123(06)53018-3 16876583

[B233] Victor T. A. Furey M. L. Fromm S. J. Ohman A. Drevets W. C. (2013). Changes in the neural correlates of implicit emotional face processing during antidepressant treatment in major depressive disorder. Int. J. Neuropsychopharmacol. 16, 2195–2208. 10.1017/S146114571300062X 23809145

[B234] Vigouroux A. (1890). Etude sur la Résistance éLectrique Chez les Mélancholiques. Thése de Paris, J. Rueff and Cie.

[B235] Wacker J. Dillon D. G. Pizzagalli D. A. (2009). The role of the nucleus accumbens and rostral anterior cingulate cortex in anhedonia: integration of resting EEG, fMRI, and volumetric techniques. Neuroimage 46, 327–337. 10.1016/j.neuroimage.2009.01.058 19457367 PMC2686061

[B236] Wager T. D. Atlas L. Y. Lindquist M. A. Roy M. Woo C. W. Kross E. (2013). An fMRI-based neurologic signature of physical pain. N. Engl. J. Med. 368, 1388–1397. 10.1056/NEJMoa1204471 23574118 PMC3691100

[B237] Ward N. G. Doerr H. O. (1986). Skin conductance. A potentially sensitive and specific marker for depression. J. Nerv. Ment. Dis. 174, 553–559. 10.1097/00005053-198609000-00008 3746283

[B238] Ward N. G. Doerr H. O. Storrie M. C. (1983). Skin conductance: a potentially sensitive test for depression. Psychiatry Res. 10, 295–302. 10.1016/0165-1781(83)90076-8 6583718

[B239] Williams K. M. Iacono W. G. Remick R. A. (1985). Electrodermal activity among subtypes of depression. Biol. Psychiatry 20, 158–162. 10.1016/0006-3223(85)90075-7 3970996

[B240] Williams L. E. Bargh J. A. (2008). Experiencing physical warmth promotes interpersonal warmth. Science 322, 606–607. 10.1126/science.1162548 18948544 PMC2737341

[B241] Williams L. E. Huang J. Y. Bargh J. A. (2009). The scaffolded mind: higher mental processes are grounded in early experience of the physical world. Eur. J. Soc. Psychol. 39, 1257–1267. 10.1002/ejsp.665 20046813 PMC2799930

[B242] Wilson A. D. Golonka S. (2013). Embodied cognition is not what you think it is. Front. Psychol. 4:58. 10.3389/fpsyg.2013.00058 23408669 PMC3569617

[B243] Yoshida M. Takayanagi Y. Inoue K. Kimura T. Young L. J. Onaka T.. (2009). Evidence that oxytocin exerts anxiolytic effects via oxytocin receptor expressed in serotonergic neurons in mice. J. Neurosci. 29, 2259–2271. 10.1523/JNEUROSCI.5593-08.2009 19228979 PMC6666325

[B244] Zahn T. P. Nurnberger J. I. Jr. Berrettini W. H. (1989). Electrodermal activity in young adults at genetic risk for affective disorder. Arch. Gen. Psychiatry 46, 1120–1124. 10.1001/archpsyc.1989.01810120062010 2589926

[B245] Zald D. H. McHugo M. Ray K. L. Glahn D. C. Eickhoff S. B. Laird A. R. (2014). Meta-analytic connectivity modeling reveals differential functional connectivity of the medial and lateral orbitofrontal cortex. Cereb. Cortex 24, 232–248. 10.1093/cercor/bhs308 23042731 PMC3862271

[B246] Zhang W. Schneider D. M. Belova M. A. Morrison S. E. Paton J. J. Salzman C. D. (2013). Functional circuits and anatomical distribution of response properties in the primate amygdala. J. Neurosci. 33, 722–733. 10.1523/JNEUROSCI.2970-12.2013 23303950 PMC3596257

[B247] Zhang X. Davidson S. Giesler G. J. Jr. (2006). Thermally identified subgroups of marginal zone neurons project to distinct regions of the ventral posterior lateral nucleus in rats. J. Neurosci. 26, 5215–5223. 10.1523/JNEUROSCI.0701-06.2006 16687513 PMC6674258

[B248] Zhong C. B. Leonardelli G. J. (2008). Cold and lonely: does social exclusion literally feel cold? Psychol. Sci. 19, 838–842. 10.1111/j.1467-9280.2008.02165.x 18947346

[B249] Ziegler D. R. Edwards M. R. Ulrich-Lai Y. M. Herman J. P. Cullinan W. E. (2012). Brainstem origins of glutamatergic innervation of the rat hypothalamic paraventricular nucleus. J. Comp. Neurol. 520, 2369–2394. 10.1002/cne.23043 22247025 PMC4440805

